# Supplementary material for: Global dynamics of microbial communities emerge from local interaction rules
Source: PLoS Comput Biol. 2022 Mar 4;18(3):e1009877. doi: 10.1371/journal.pcbi.1009877 (PMC8926250; doi:10.1371/journal.pcbi.1009877)
Supplement: S1 Text — Here we derive the local interactions rules for 2D and 3D mutualistic communities from the molecular parameters underlying the interaction using a biophysical model. Fig A: The interaction range is similar for 2D and 3D communities and closely matches the analytical prediction for the growth range. (PDF) [file pcbi.1009877.s001.pdf]

S1 Text

Local interaction rules for cross-feeding communities

Contents

1 Local interaction rules for two-dimensional communities 1

2 Local interaction rules for three-dimensional communities 3

3 Local interaction rules for communities consisting of dissimilar cell types 5

4 Estimating neighborhood size from interaction range 9

5 References 10

1 Local interaction rules for two-dimensional communities

We previously showed that the local interaction rules for cross-feeding communities can be fully specified with two quantities: the range over which cells can obtain nutrients, i.e. the interaction range R, and the maximum rate at which cells grow when they are fully surrounded by the partner type, $\hat{\mu}$ [1]. Using a biophysical model, we showed that these two quantities can be derived from the biophysical parameters underlying the exchange of metabolites. We previously derived these results assuming that communities grow in two-dimensional structures and consist of closely related cell types that share the same pathways for the uptake and release of nutrients. Here we will generalize our biophysical model by extending it to three-dimensional communities, and to communities consisting of cell types that can differ substantially in their pathways for the uptake and release of nutrients. The extended model presented here is thus generally applicable to any cross-feeding community consisting of two cell types.

Summary of previous results for two-dimensional communities Here, we will briefly summarize the local rules for two-dimensional cross-feeding communities which we derived previously, for the full derivation we refer the reader to the supplementary information of reference [1].

1.1 Interaction range

The first essential parameter that describes the local rules of cross-feeding communities is the interaction range R, which specifies the distance, measured from the cell surface, over which

amino-acids are primarily exchanged. We previously derived an analytical approximation for this interaction range based on the molecular parameters of the underlying nutrient exchange [1]. Specifically, for simple spatial arrangements where the two cell types are separated by a straight interface, we could calculate how the growth rate decreases away from the interface between the two cell types. We calculated the distance over which the growth rate decreases by 50% (i.e. the growth range) and showed that this distance is directly proportional to the interaction range; the interaction range is a measure of the distance over which amino acids can be exchanged in an arbitrarily complex spatial arrangement [1]. We found that the interaction range can be approximated as:

$$R = \beta \sqrt{\frac{2(1-\rho)^2 \cdot D}{\rho(2+\rho) \cdot (r^u + r^l)}} \ln \left[\frac{r^l}{\gamma} \left(1 + \sqrt{1 + \frac{4\gamma}{r^l}} \right) + 4 \right] \tag{1}$$

where β and γ are constants, ρ is the 3D cell density (i.e. the volume fraction occupied by cells), and D, r^u, and r^l are the rates of diffusion, uptake, and leakage of the exchanged metabolite. The square root term can be interpreted as the distance that a molecule travels before it is taken up by a cell, and this depends primarily on the ratio of diffusion rate and uptake rate and the cell density. The constant β is the constant of proportionality between the growth range and the interaction range, and we previously showed that $\beta \approx 0.88$ [1].

The constant $\gamma = 2\mu^{wt}/\mathcal{I}^C$ is species specific, but does not depend on the properties of the exchanged molecules. Here, μ_{wt} is the maximum growth rate of a wild type cell that can produce all essential metabolites. $\mathcal{I}^C = I^C/K_M$ is the internal concentration of the essential metabolite in a producing cell (I^C) relative to the Monod constant of the growth curve (K_M; growth is assumed to follow Monod kinetics as function of the internal concentration (I) of the essential metabolite: $\mu(I) = \frac{\mu_{wt} I}{I + K_M}$). The factor $\frac{r^l}{\gamma} = \frac{r^l I^C}{2\mu^{wt} K_M}$ can be interpreted as the flux of metabolites leaked into the environment, relative to the flux of metabolites used for growth. It is thus a measure of leakiness: if it is close to 1, a large fraction of the essential metabolite is lost to the environment, while if it is much smaller than 1 most of the essential metabolite is kept within the cell and used for growth. To derive Eq. 1 we assumed that $r^l \ll \mu_{wt}$ and $I^C \gg K_M$. These assumptions thus state that cells have limited leakiness. We previously showed that these assumptions are compatible with the measurements from an experimental cross-feeding community [1].

1.2 Maximum growth rate

The second essential parameter that describes the local rules of cross-feeding communities is the maximum growth rate that cells can obtain when they are fully surrounded by the other cell type, $\hat{\mu}$. We previously showed that [1]:

$$\hat{\mu} \approx \mu_{wt} \cdot \frac{r^l}{\gamma} \left(\sqrt{1 + \frac{2\gamma}{r^l}} - 1 \right) \tag{2}$$

2 Local interaction rules for three-dimensional communities

The analytical expressions for the local interaction rules described above were originally derived for two-dimensional systems, however we will show here that they also hold for three-dimensional systems. Specifically, the derivation for the maximum growth rate (Eq. 2) only assumed that isolated non-producing cells, fully surrounded by producing cells do not substantially change the external concentration of the exchanged compound; this assumption holds equally in 2D and 3D. The analytical expression for the growth range (Eq. 1) also generalizes to 3D. The primary assumption we made to derive this result is that the interface between the two cell types is quasi-1D; in 2D systems this assumption means the two types are separated by a straight line, and in 3D it means the two types are separated by a plane. For these very simple configurations we can then calculate the growth range using the same quasi-1D approximation. Even though the growth range R is identical for 2D and 3D systems, the neighborhood size r does depend on dimensionality. For spherical cells, the number of cells within a distance R of a central cell is proportional to R^2 in 2D and proportional to R^3 in 3D. For rod-like cells the relation between the number of neighbors r and the growth range R is more complicated, as we will show below (Eq. 21 for 2D and 22 for 3D), however it is still true that the number of neighbors, at a constant growth range, is higher in 3D than in 2D systems.

For more complex spatial arrangements we cannot calculate the growth range analytically, however we can use an individual based model that we developed previously to numerically calculate the interaction range for any arbitrary 2D spatial arrangement [1]. Here we extended this individual based model to 3D. This is done by replacing the square 2D grid with a cubic 3D grid, and by using a 3D instead of 2D Laplacian to calculate the diffusion of metabolites (see Eq. 15 and 16 in the SI of reference [1]). We used this model to calculate the interaction range for 2D and 3D communities with varying spatial arrangements. The grid is initialized by randomly assigning each grid point to one of the two cell types. To vary the patch size, we pick a random cell and let it replace one of its randomly chosen neighbors; for each *replication cycle* this is done until all cells on the grid have replaced one of their neighbors. The procedure can be repeated for more cycles to achieve larger patches on average (see Fig A-A). For each grid we then calculate the steady state growth rates of all cells, and then use these growth rates to calculate the interaction range using the correlation method we previously developed. In short: the growth rate of a cell is correlated with the frequency of the partner type within a given radius. The interaction range is defined as the radius at which this correlation is maximal (see reference [1] for details).

Using the individual based model we can show that the interaction range we derived is very similar for 2D and 3D systems. Fig A-B shows the calculated interaction range for 2D and 3D arrangements of a symmetric community for varying patch sizes. The estimated interaction ranges are very similar between 2D and 3D systems, and both are close to the analytically

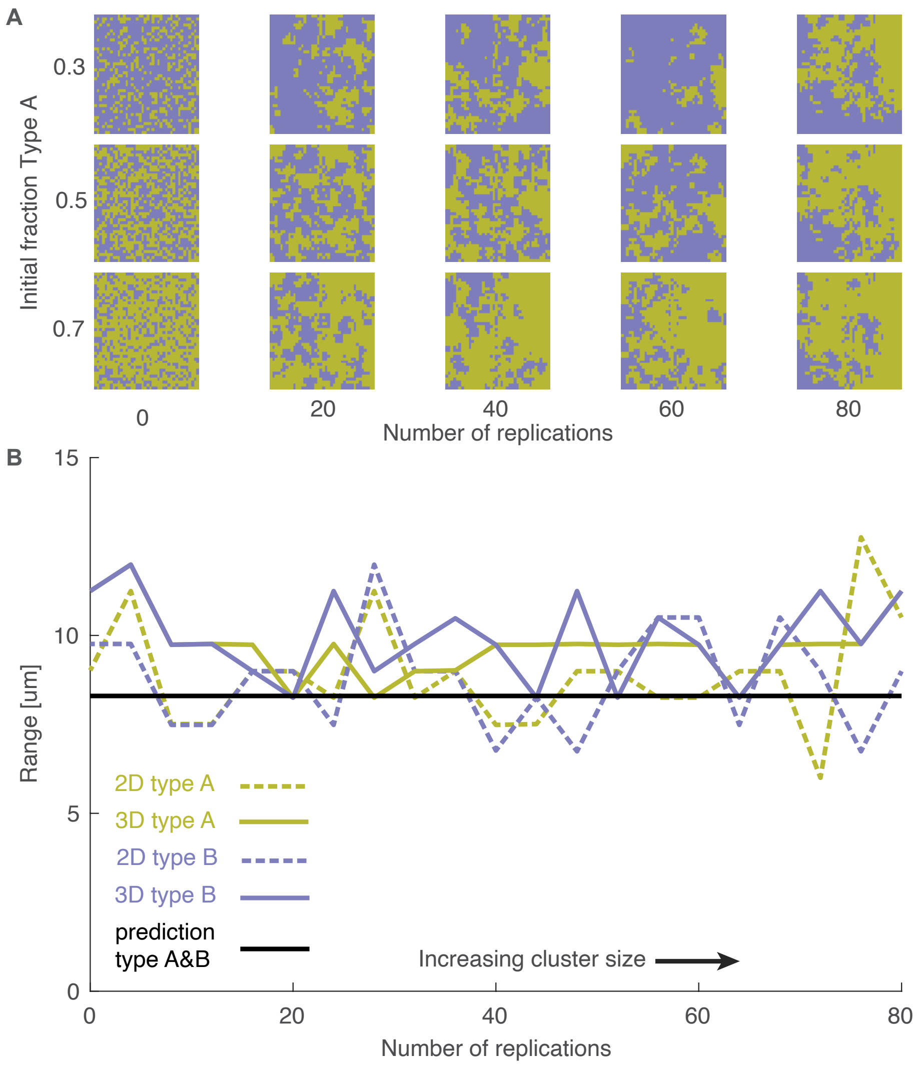

Fig A. The interaction range is similar for 2D and 3D communities and closely matches the analytical prediction for the growth range. The interaction range was calculated based on in-silico data for a community consisting of two cell types (A and B) with identical molecular rates. To calculate the interaction range, we used a previously developed individual based model [1] to predict the growth rate of all cells growing either in a 2D or 3D community; then used a correlation method [1] to estimate the interaction range for both cell types. The interaction range is compared to the growth range, which is calculated analytically from Eq. 1. For each parameter set, we simulated 100 communities of 40x40 cells (2D) or 40x40x40 cells (3D), with closed (no-flux) boundaries on all sides as described previously [1]. The grids were seeded by randomly assigning the two cell types with a frequency of type A between 0.2 and 0.8. To implement larger patches we allowed all cells (in random order) to replace one of their neighbors (chosen at random). In total we performed 0 to 80 of these replication cycles. The more replication cycles the larger the average patch size. A) Typical spatial arrangements as function of the initial frequency of type A (y-axis) and the number of replication cycles (x-axis), the central slice of the 3D grid is shown. B) The calculated interaction range is shown as function of the number of replication cycles (i.e., patch size) and compared to the analytical prediction for the growth range. Parameters: $r_A^u = r_B^u = 3.58\ 1/s$, $r_A^l = r_B^l = 3.58 \cdot 10^{-6}\ 1/s$, $D_A = D_B = 7.17 \cdot 10^2\ \mu m^2/s$, all other parameters as in S1 Table.

calculated growth range (Eq. 1). This thus shows that our estimate of the range over which cell can interact holds for both 2D and 3D systems.

3 Local interaction rules for communities consisting of dissimilar cell types

Previously we assumed that uptake and leakage rates differ between chemical compounds, but not between cell types [1]. This assumption holds whenever all cell types share the same pathway for uptake and leakage, as it is the case for microbial communities that consist of closely related strains or for different cell types in a multicellular organism. However, in general uptake and leakage rates could differ both between chemical compounds and between cell types, due to difference in uptake and leakage pathways. Here we will derive the local rules, i.e. the maximum growth range and interaction range, for such systems. We closely follow reference [1] to derive these quantities and we refer the reader to that document for more details on the derivation.

We track the internal I and external E concentration of the exchanged compounds. For notational simplicity, we focus on the exchange of a single compound and we write down the equations for the internal I_p and external E_p concentration for cells that can produce this compound:

$$\frac{\partial I_p}{\partial t} = 0 \tag{3}$$

$$\frac{\partial E_p}{\partial t} = -\alpha \cdot r_p^u \cdot E_p + \alpha \cdot r_p^l \cdot (I_p - E_p) + D^{eff} \nabla^2 E_p \tag{4}$$

where r_p^u and r_p^l are the uptake and leakage rates of the exchanged compound of the producing cells, respectively, and where $D^{eff} = \frac{(1-\rho) \cdot D}{(1+\frac{\rho}{2})}$ is the effective diffusion constant in a crowded environment, and where $\alpha = \frac{V_{in}}{V_{out}} = \frac{\rho}{1-\rho}$ is the ratio between the intra- and extracellular volume. Similarly, we write the equations for the internal I_n and external E_n concentration for cells that cannot produce this compound:

$$\frac{\partial I_n}{\partial t} = r_n^u \cdot E_n - r_n^l \cdot (I_n - E_n) - \frac{\mu_n \cdot I_n}{K_n + I_n} \cdot I_n \tag{5}$$

$$\frac{\partial E_n}{\partial t} = -\alpha \cdot r_n^u \cdot E_n + \alpha \cdot r_n^l \cdot (I_n - E_n) + D^{eff} \nabla^2 E_n \tag{6}$$

where r_n^u and r_n^l are the uptake and leakage rates of the exchanged compound of the non-producing cells, respectively, K_n is the Monod constant for the non-producing cells (the concentration at which cells can grow at half-maximum rate), and μ_n is the growth rate that auxotrophic cells can reach when the exchanged compound is non-limiting.

By solving Eq. 5 for the temporal steady state we can find an expression for the internal concentration as function of the external concentration inside consumer cells:

$$I_n(E_n) = \frac{(r_n^u + r_n^l)E_n - r_n^l K_n + \sqrt{((r_n^u + r_n^l)E_n + r_n^l K_n)^2 + 4(r_n^u + r_n^l)\mu_n K_n E_n}}{2(\mu_n + r_n^l)} \tag{7}$$

3.1 Maximum growth rate

Here we derive the analytical expression for the growth rate of a non-producing cell surrounded by a large number of producing partners. We assume that the single non-producing cell has a negligible influence on the external concentration of the exchanged molecules; thus this concentration is identical to that in an area fully occupied by producers cells and can be found by solving Eq. 3 and 4 at steady state :

$$E^{max} = \frac{r_p^l}{r_p^u + r_p^l} \cdot I_p^C \tag{8}$$

where I_p^C is the internal concentration of the produced compound inside the producer cell. We can then calculate the growth rate of the consumer cell using:

$$\mu^{max} = \frac{\mu_n I_n(E^{max})}{K_n + I_n(E^{max})} \tag{9}$$

where $I_n(E^{max})$ is found by substituting E_n in Eq. 7 with E^{max} as given by Eq. 8. Although an analytical expression can be obtained, it is rather complex and we therefore follow reference [1] and further simplify the expression by assuming:

Assumption 1 $\frac{I_p^C}{K_n} \gg \frac{r_n^l}{r_p^l} \cdot \frac{r_p^u + r_p^l}{r_n^u + r_n^l}$. *This is identical to Assumption 1 in reference [1], with the additional requirement that the difference in uptake and leakage rates between the two cell types is not too large.*

Assumption 2 $r^l \ll \mu^{aux}$. *This is identical to Assumption 2 in reference [1].*

Using these assumptions we find the following expression for the maximum growth that non-producing cells can obtain in a cross-feeding consortium:

$$\mu^{max} \approx \mu_n \cdot \theta \left(\sqrt{1 + \frac{2}{\theta}} - 1 \right) \tag{10}$$

$$\theta = \frac{r_p^l I_p^C}{2\mu_n K_n} \cdot \frac{r_n^u + r_n^l}{r_p^u + r_p^l} \tag{11}$$

The first term in the constant θ measures the leakage flux in producing cells ($r_p^l I_p^C$) relative to the flux needed by non-producing cells to grow well ($2\mu_n K_n$). The second term corresponds to the

effective uptake rate (active transport with rate r_n^u together with diffusion across the membrane with rate r_n^l) in non-producing cells relative to that in producing cells. In the case where cell types have identical rates, $\theta = \frac{r^l I^C}{2\mu K} = \frac{r^l}{\gamma}$ and we thus recover Eq. 2 (Eq. 25 in SI of reference [1]) which we previously derived for communities where both cell types have the same uptake and leakage rates. Eq. 10 increases monotonically with θ showing that the maximum growth rate of non-producing cells increases with the leakage rate of producer cells (r_p^l) and the uptake rate of non-producing cells (r_n^u), while it decreased with the uptake rate of producer cells (r_p^u).

3.2 Growth range

Next we will re-derive the growth range for communities in which the cell types differ in their uptake and leakage rates. The derivation closely follows reference [1]. We consider a scenario where the two cell types are symmetrically arranged i.e. when the two cell types are separated by a straight line in 2D or by a flat plane in 3D. This reduces the problem to one-dimension, x, which measures the distance of a cell to the interface. Producing cells are located at $x < 0$ and non-producing cells at $x > 0$. We will here derive an analytical approximation for the growth rate of the non-producing cells by setting Eq. 4 and 6 to steady state:

$$\frac{d^2E}{dx^2} = \begin{cases} \frac{1}{r_{p0}^2}\left(E - \frac{r_p^l}{r_p^l + r_p^u} I_p^C\right) & \text{if } x < 0 \\ \frac{1}{r_{n0}^2}\left(E - \frac{r_p^l}{r_p^l + r_p^u} I_n(E)\right) & \text{if } x > 0 \end{cases} \tag{12}$$

where

$$r_{p0} = \sqrt{\frac{D^{eff}}{\alpha(r_p^u + r_p^l)}} \qquad r_{n0} = \sqrt{\frac{D^{eff}}{\alpha(r_n^u + r_n^l)}}$$

For $x > 0$ the analytical solution of Eq. 12 cannot be found due to the non-linear term $I_n(E)$ (given by Eq. 7), however we previously showed that this term can be ignored close to the interface [1]. We thus solve the following approximate ODE:

$$\frac{d^2E}{dx^2} = \begin{cases} \frac{1}{r_{p0}^2}\left(E - \frac{r_p^l}{r_p^l + r_p^u} I_p^C\right) & \text{if } x < 0 \\ \frac{1}{r_{n0}^2} E & \text{if } x > 0 \end{cases} \tag{13}$$

This equation can be solved analytically to find:

$$E(x) = \begin{cases} C_1 \cdot e^{x/r_0 p} + \frac{r_p^l}{r_p^l + r_p^u} I_p^C & \text{if } x < 0 \\ C_2 \cdot e^{-x/r_0 n} & \text{if } x > 0. \end{cases}$$

We can solve for C_1 and C_2 by imposing continuity of concentration and flux at the interface and find:

$$E(x) = \begin{cases} \frac{I_p^C r_p^l \left(r_{0n} - r_{0p} e^{\frac{x}{r_{0p}}} + r_{0p}\right)}{\left(r_p^l + r_p^u\right)\left(r_{0n} + r_{0p}\right)} & \text{if } x < 0 \\ \frac{I_p^C r_p^l r_{0n} e^{-\frac{x}{r_{0n}}}}{\left(r_p^l + r_p^u\right)\left(r_{0n} + r_{0p}\right)} & \text{if } x > 0 \end{cases} \tag{14}$$

We now calculate an analytical approximation for the *growth range* (R), which is the distance from the interface where cells have 50% of the growth rate they have at the interface:

$$\mu(x = R) = \frac{1}{2} \cdot \mu(x = 0) \tag{15}$$

as $\mu = \frac{\mu_n \cdot I_n}{K_n + I_n}$ it follows that

$$I_n\Big|_{x=R} = \frac{I_n\Big|_{x=0}}{2 + I_n\Big|_{x=0}} \tag{16}$$

where $I_n\Big|_x \equiv I_n(E(x))$. Substituting Eq. 7 for $I_N(E)$ and Eq. 14 for $E(x)$ we can thus solve Eq. 16 for the growth range. To convert the growth range to the interaction range R, we can simply multiply with the constant $\beta \approx 0.88$:

$$R = \beta \cdot r_{0n} \log \left[\frac{\left(4r_n^l - \delta r_p^l\right)\left(\sqrt{2\delta r_p^l \mu_n + \left(\frac{\delta r_p^l}{2} + r_n^l\right)^2} + \frac{\delta r_p^l}{2} + r_n^l\right) - 4\delta r_p^l \left(\mu_n + r_n^l\right)}{2r_n^l \left(2r_n^l - \delta r_p^l\right) - \delta r_p^l \mu_n} \right] \tag{17}$$

where

$$\delta = \frac{r_p^l I_p^C}{2\mu_n K_n} \cdot \frac{2r_{0n}}{r_{0n} + r_{0p}} \cdot \frac{r_n^u + r_n^l}{r_p^u + r_p^l} \tag{18}$$

$$= \frac{r_p^l I_p^C}{2\mu_n K_n} \cdot \frac{2\sqrt{r_p^u + r_p^l}}{\sqrt{r_n^u + r_n^l} + \sqrt{r_p^u + r_p^l}} \cdot \frac{r_n^u + r_n^l}{r_p^u + r_p^l} \tag{19}$$

The first term in the constant δ measures the leakage flux in producing cells ($r_p^l I_p^C$) relative to the flux needed by non-producing cells to grow well ($2\mu_n K_n$). The second term corresponds to the diffusion length scale r_{0n} in regions occupied by non-producing cells, relative to the average

diffusion length scale $(r_{0p} + r_{0n})/2$. The third term corresponds to the effective uptake rate (active transport with rate r_n^u together with diffusion across the membrane with rate r_n^l) in non-producing cells relative to that in producing cells.

Using assumption 2 and

Assumption 3 $\frac{I_p^C}{K_n} \gg \frac{r_n^l}{r_p^l} \cdot \frac{r_{0n}+r_{0p}}{r_{0n}} \cdot \frac{r_p^u+r_p^l}{r_n^u+r_n^l}$. *This is identical to Assumption 1 in reference [1], with the additional requirement that the difference in uptake and leakage rates between the two cell types is not too large.*

this can be simplified to:

$$R \approx \beta \sqrt{\frac{D^{eff}}{\alpha(r_n^u + r_n^l)}} \cdot \ln\left[\delta\left(1 + \sqrt{1 + \frac{4}{\delta}}\right) + 4\right] \tag{20}$$

In communities where both cell types have the same uptake and leakage rates, $\delta = \frac{I^C r^l}{2K\mu} = \frac{r^l}{\gamma}$, recovering our previous results (i.e. Eq. 1). Eq. 20 is a monotonically decreasing function of r_n^u, so the higher the uptake rate of the consumer, the lower the growth range. When r_n^u and r_n^l are held constant, Eq. 20 is a monotonically increasing function of δ.

4 Estimating neighborhood size from interaction range

Above we derived the local rules for cross-feeding systems by calculating the maximum growth range and the interaction range of cells. The interaction range determines the interaction neighborhood of a cell by specifying the maximum distance, measured from surface of a cell, over which cells can obtain nutrients. However, in the pair-approximation framework we derive in S2 Text the interaction neighborhood has to be specified as the number of cells that are contained within it. These two metrics, i.e. the interaction range, measured in units of distance, and the neighborhood size, measured as a number of cells, can be converted into each other using a simple geometrical calculation.

For two-dimensional systems, we can estimate the number of neighbors (r, measured in number of cells) from the interaction range (R, measured in units of length) using measured values of the cell geometry and the cell area density ρ_{2D} (i.e. the number of cells per area). We assume that cells (as seen in a 2D projection) can be described as rectangles with semi-spherical end-caps, with total length l and width w. The number of cells within the interaction neighborhood (i.e. within the area within distance R from the cell-surface) is then given by:

$$r = \left(2R(l-w) + \pi\left(R + \tfrac{w}{2}\right)^2 - \pi\left(\tfrac{w}{2}\right)^2\right) \cdot \rho_{2D} \tag{21}$$

We can make a similar estimate for three-dimensional systems. We assume that cells can be described as cylinders with semi-spherical end-caps, with total length l and width w. The

number of cells within the interaction neighborhood (i.e. within the area within distance R from the cell surface) is then given by:

$$r = \frac{\left(R(R+w)(l-w) + \frac{4}{3}\left(\left(R+\frac{w}{2}\right)^3 - \left(\frac{w}{2}\right)^3\right)\right) \cdot \rho}{(l-w)(\frac{w}{2})^2 + \frac{w^3}{6}} \tag{22}$$

5 References

1. Dal Co, A., van Vliet, S., Kiviet, D.J., Schlegel, S. & Ackermann, M. Short-range interactions govern the dynamics and functions of microbial communities. *Nat Ecol Evol* **4**, 366–375 (2020).
