# Supplementary material for: Global dynamics of microbial communities emerge from local interaction rules
Source: PLoS Comput Biol. 2022 Mar 4;18(3):e1009877. doi: 10.1371/journal.pcbi.1009877 (PMC8926250; doi:10.1371/journal.pcbi.1009877)
Supplement: S2 Text — Here we derive the community level properties from the local interaction rules by applying pair-approximation to a graph based model. (PDF) [file pcbi.1009877.s002.pdf]

# S2 Text

## Predicting community level dynamics from local rules using pair-approximation

## Contents


**1 Model derivation** **1**

1.1 Definitions . . . 1

1.2 Pair-approximation . . . 2

1.3 Cross-feeding communities . . . 5

1.4 Arbitrary communities of two interacting cell types . . . 10

**2 Model application** **11**

2.1 Cross-feeding interaction . . . 11

2.2 Density dependent interaction . . . 12

2.3 Growth inhibition . . . 13

2.4 Application to an experimental cross-feeding community . . . 13

**3 References** **14**


# 1 Model derivation

We model a community consisting of two interacting cell types, $A$ and $B$, using evolutionary graph theory. In contrast to previous work, we allow the interaction neighborhoods to be different for the two types. As a result interactions are not symmetric, and we need to use directed graphs. We extend traditional pair approximation to find analytical expressions for the dynamics and the equilibrium state of the community. Pair approximation assumes that the spatial arrangements of any system can be described by only tracking pairwise correlations. As a consequence, the system can be fully described by tracking the number of all possible $(X \leftarrow Y)$ pairs. Pair approximation neglects any stochastic effects arising from finite populations and from finite numbers of cells in the interaction neighborhood.

## 1.1 Definitions

We consider two cell types, $A$ and $B$, and use the notation $Y \in \{A, B\}$ to indicate cell types. Cells are placed on the vertices of a directed graph. The total number of cells, $N$, is constant in time. At any given time, there are $N_A(t)$ and $N_B(t)$ cells of type $A$ and $B$, respectively. For notational simplicity, we will not explicitly show time dependence in our variables and simply write $N_A$ and $N_B$. Unless explicitly noted otherwise, all variable are time-dependent.

Interactions are encoded by directed links; in our notation $X \leftarrow Y$ indicates that focal cell $X$ interacts with neighboring cell $Y$, i.e. cell $X$ obtains metabolites from cell $Y$. All type $A$ cells interact with $r_A$ individuals, and we call the set of all neighbors with which a given $A$ cell interacts the small interaction neighborhood $I_A$. All type $B$ cells interact with $r_B$ individuals, and we call the set of all neighbors with which a given $B$ cell interacts the large interaction neighborhood $I_B$. Without loss of generality, we assume that type $A$ has the smallest interaction neighborhood, i.e. $r_A \leq r_B$. We also assume that the small interaction neighborhood is contained within the large one: $I_A \subseteq I_B$. This assumption comes from a simple geometric consideration: for spherical cells the interaction neighborhood is given by a circle/sphere (in 2D/3D space, respectively) centered on the focal individual with an area/volume that is set by the neighborhood size. The smaller interaction neighborhood is thus always fully contained within the larger one. More generally, for non-spherical cells the interaction neighborhood follows the shape of the focal cell and is defined as the region of space within a fixed distance from the cell-surface such that the total number of cells contained in this regions equals the size of the neighborhood. Cells within the smaller interaction neighborhood are those neighbors that are closest to the focal cell; these cells are thus also members of the larger interaction neighborhood (which extends further away from the focal cell).

Because the neighborhood sizes of the two types are different, links are not symmetric, i.e. if there is a link $X \leftarrow Y$ between two cells, this does not imply that there is also a link $Y \leftarrow X$. The number of $Y \leftarrow X$ links is given by $N_{Y \leftarrow X}$. The total number of links in the system $L$ varies over time, because type $A$ and $B$ have different neighborhood sizes and the relative frequency of $A$ and $B$ changes in time:

$$L = N_{A\leftarrow A} + N_{A\leftarrow B} + N_{B\leftarrow A} + N_{B\leftarrow B} \tag{1}$$

Cells can place offspring within the replication neighborhood; the set of all neighbors that can be replaced is denoted by $I_R$, and counts $r_R$ cells. For mathematical tractability, we assume that the replication neighborhood is identical to smallest interaction neighborhood, i.e. $I_R = I_A$.

For each focal cell $X$, independently of its type, we can define two additional neighborhoods: 1) the small neighborhood $S(X, r_A)$ which consists of $r_A$ cells. This is the set of cells that can be replaced by the focal cell $X$ ($S(X, r_A) = I_R$). When we consider a focal cell of type $A$, this set of cells consists of all cells with which the $A$ focal cell interacts ($S(A, r_A) = I_A$). 2) the large neighborhood $S(X, r_B)$ which consists of $r_B$ cells. When we consider a focal cell of type $B$, this

set of cells consists of all cells with which the $B$ focal cell interacts ($S(B, r_B) = I_B$). We assume $S(X, r_A) \subseteq S(X, r_B)$.

Throughout, we will use capital $N$ to denote numbers within the entire system, and small $n$ to denote numbers corresponding to the local neighborhood of a focal cell. For example, $N_A$ is the total number of $A$ cells in the system, while $n_A$ is the number of $A$ cells in the neighborhood of the focal cell.

## 1.2 Pair-approximation

The system can be described by tracking the number of pairwise links $N_{X \leftarrow Y}$. We can express the total number of type $A$ and $B$ cells as function of $N_{X \leftarrow Y}$:

$$N_A = \frac{N_{A \leftarrow A} + N_{A \leftarrow B}}{r_A}, \quad N_B = \frac{N_{B \leftarrow A} + N_{B \leftarrow B}}{r_B} \tag{2}$$

each type $A$ focal cell has $r_A$ incoming links, which originate either from a type $A$ neighbor ($N_{A \leftarrow A}$ times) or from a type $B$ neighbor ($N_{A \leftarrow B}$ times). The number of $A$ cells $N_A$ can be found dividing the total number of incoming links over all $A$ cells by the number of incoming links per single $A$ cell. A similar reasoning allows to calculate the number of $B$ cells.

Because the total number of cells in the system ($N = N_A + N_B$) is constant, it follows from Eq. 2 that:

$$\frac{N_{A \leftarrow A} + N_{A \leftarrow B}}{r_A} + \frac{N_{B \leftarrow A} + N_{B \leftarrow B}}{r_B} = N = \text{constant} \tag{3}$$

We thus have three independent variables: one of the four quantities $N_{X \leftarrow Y}$ can be expressed as function of the other three.

To describe the composition of the system, we need the probability that a random cell we pick in the system is of either type $A$ or $B$. These probability, $P(A)$ and $P(B)$, follow directly from Eq. 2:

$$P(A) = \frac{N_{A \leftarrow A} + N_{A \leftarrow B}}{r_A N}, \quad P(B) = \frac{N_{B \leftarrow A} + N_{B \leftarrow B}}{r_B N} = 1 - P(A) \tag{4}$$

Moreover, we need to know the conditional probabilities that describe the local neighborhood of a cell. We define $P(Y|X, r_A)$ as the probability to find a neighbor of type $Y$ within the small neighborhood (i.e. in the set $S(X, r_A)$), given that the focal cell is of type $X$. Likewise, $P(Y|X, r_B)$ is the probability to find a neighbor of type $Y$ within the large neighborhood (i.e. in the set $S(X, r_B)$), given that the focal cell is of type $X$.

The conditional probabilities $P(Y|A, r_A)$ describe the average composition of the small neighborhood surrounding any type $A$ focal cell. By definition, the small neighborhood is identical to the interaction neighborhood of a type $A$ cell, $S(A, r_A) = I_A$. All cells in this small neighborhood, and only these cells, interact with the focal $A$ cell. All these cells are thus connected to the focal cells by $A \leftarrow Y$ links. From the number of $A \leftarrow Y$ links, we can directly calculate the

conditional probabilities $P(Y|A, r_A)$:

$$P(A|A, r_A) = \frac{N_{A \leftarrow A}}{N_{A \leftarrow A} + N_{A \leftarrow B}}, \quad P(B|A, r_A) = 1 - P(A|A, r_A)$$

The equation for $P(A|A, r_A)$ can be intuitively understood as follows: the probability of finding a $A$ neighbor within the interaction neighborhood of an $A$ focal cell is simply the fraction of incoming links that start from $A$ cells.

Likewise, we can directly calculate the conditional probabilities $P(Y|B, r_B)$ from the number of $B \leftarrow Y$ links, because the large neighborhood is identical to the interaction neighborhood of type $B$, i.e. $S(B, r_B) = I_B$. We thus find:

$$P(A|B, r_B) = \frac{N_{B \leftarrow A}}{N_{B \leftarrow A} + N_{B \leftarrow B}}, \quad P(B|B, r_B) = 1 - P(A|B, r_B)$$

In contrast, the conditional probabilities $P(Y|A, r_B)$ and $P(Y|B, r_A)$ cannot be directly calculated from the pairwise links. $P(Y|A, r_B)$ describes the composition of the large neighborhood surrounding a type $A$ focal cell. This neighborhood can be larger than the interaction neighborhood of type $A$, and as a result the focal cell does not necessarily have incoming links from all cells in the large neighborhood. In other words, the set of cells in the interaction neighborhood of the type $A$ focal cell is only a subset of all cells in the large neighborhood: $I_A \subseteq S(A, r_B)$. $P(Y|B, r_a)$ describes the composition of the small neighborhood surrounding a type $B$ focal cell. The small neighborhood can be smaller than the interaction neighborhood of the type $B$ focal cell, i.e. $S(B, r_A) \subseteq I_B$. As a result, links to the type $B$ focal cell are not exclusive to the small neighborhood.

We can find the conditional probabilities $P(Y|A, r_B)$ and $P(Y|B, r_A)$ using a requirement of self-consistency. Suppose that we want to calculate the average number of type $A$ cells in the community. We can calculate this number directly from the global probability of finding a type $A$ cell as $N_A = P(A)N$. Alternatively, we can visit each cell in the community and calculate the average number of type $A$ neighbors it has. We will first do this considering the large $S(X, r_B)$ neighborhood for each cell. In $P(A)N$ cases the focal cell is of type $A$, which has $P(A|A, r_B)r_B$ type $A$ neighbors. In $P(B)N$ cases the focal cell is of type $B$, which has $P(A|B, r_B)r_B$ type $A$ neighbors. Each neighboring cell is counted $r_B$ times (it is part of the large $S(X, r_B)$ neighborhood of $r_B$ focal cells). Summing these two numbers and dividing by $r_B$ to correct for this multiple counting then gives the total number of $A$ cells in the system:

$$N_A = \frac{P(A)N \cdot P(A|A, r_B)r_B + P(B)N \cdot P(A|B, r_B)r_B}{r_B} = N\left(P(A) \cdot P(A|A, r_B) + P(B) \cdot P(A|B, r_B)\right)$$

Self-consistency requires that both methods give the same estimate for the average number of $A$ cells:

$$P(A)N = N\left(P(A)\cdot P(A|A,r_B) + P(B)\cdot P(A|B,r_B)\right)$$

From this we can thus find an expression for $P(A|A,r_B)$ as function of $P(A|B,r_B)$:

$$P(A|A,r_B) = 1 - \frac{P(B)}{P(A)}P(A|B,r_B)$$

We can repeat this procedure and count the number of $A$ and $B$ cells in the neighborhood of any $X$ cell, both using the small $S(X,r_A)$ and large $S(X,r_B)$ neighborhoods, to find expressions for the other undefined conditional probabilities $P(Y|A,r_B)$ and $P(Y|B,r_a)$. We can fully describe the local arrangements of the spatial system using the following set of conditional probabilities:

$$\begin{aligned}
P(A|A,r_A) &= \frac{N_{A\leftarrow A}}{N_{A\leftarrow A}+N_{A\leftarrow B}}, & P(A|A,r_B) &= 1-\frac{P(B)}{P(A)}\cdot P(A|B,r_B)\\
P(B|A,r_A) &= \frac{N_{A\leftarrow B}}{N_{A\leftarrow A}+N_{A\leftarrow B}}, & P(B|A,r_B) &= \frac{P(B)}{P(A)}\cdot P(A|B,r_B)\\
P(A|B,r_A) &= \frac{P(A)}{P(B)}\cdot P(B|A,r_A), & P(A|B,r_B) &= \frac{N_{B\leftarrow A}}{N_{B\leftarrow A}+N_{B\leftarrow B}}\\
P(B|B,r_A) &= 1-\frac{P(A)}{P(B)}\cdot P(B|A,r_A), & P(B|B,r_B) &= \frac{N_{B\leftarrow B}}{N_{B\leftarrow A}+N_{B\leftarrow B}}
\end{aligned} \tag{5}$$

## 1.3 Cross-feeding communities

We will first use pair-approximation to derive predictions for the community-level properties of cross-feeding communities. In the next section we will generalize these results to any arbitrary community of two interacting cell types.

### 1.3.1 Dynamical equations for community-level dynamics

We assume for now that growth rates depend linearly on the frequency of the partner type within the interaction neighborhood (we will later consider a more general growth function). Note that here we use the term growth rate to refer to the rate at which individual cells increase there biomass, e.g. it corresponds to the elongation rate of rod-shape bacteria. For microbes the growth rate is equal to the birth rate, i.e. the rate at which new cells are produced. We will thus use the terms growth rate and birth rate interchangeably.

$$\mu_A(n_B) = \frac{n_B}{r_A}\cdot\hat{\mu}_A, \quad \mu_B(n_A) = \frac{n_A}{r_B}\cdot\hat{\mu}_B \tag{6}$$

where $n_B$ is the number of type $B$ cells within the interaction neighborhood of type $A$ cell, $n_A$ is the number of type $A$ cells within the interaction neighborhood of a type $B$ cell, $\hat{\mu}_A$ and $\hat{\mu}_B$ are the maximum growth rates of type $A$ and $B$, respectively.

Two events can change the number of links: a type $A$ cell reproduces and replaces a $B$ neighbor, with rate $T^+$, or a type $B$ cell reproduces and replaces an $A$ neighbor, with rate $T^-$. During a $T^+$ event the number of type $A$ cells thus increases by one, and during a $T^-$ event it decreases by one. To calculate rate $T^+$ we need to consider all events were an $A$ cell replaces a $B$ cell. A type $A$ cell can have $0 \leq n_B < r_A$ type $B$ neighbors. The probability of finding an $A$ cells with $n_B$ B neighbors is given by:

$$P(A) \cdot P(A|A, r_A)^{r_A - n_B} \cdot P(B|A, r_A)^{n_B} \cdot \binom{r_A}{n_B}$$

The probability that this cell reproduces is proportional to its growth rate, $\mu_A(n_B)$.

$$\frac{n_B}{r_A} \cdot \hat{\mu}_A$$

And the probability that the resulting offspring replaces a type $B$ cell is given by:

$$\frac{n_B}{r_A}$$

The rate $T^+$ can be found by multiplying these probabilities, summing over all possible number of $n_B$, and multiplying with the total population size $N$:

$$T^+ = N \cdot \sum_{n_B=0}^{r_A} P(A) \cdot P(A|A, r_A)^{r_A - n_B} \cdot P(B|A, r_A)^{n_B} \cdot \binom{r_A}{n_B} \cdot \frac{n_B}{r_A} \cdot \hat{\mu}_A \cdot \frac{n_B}{r_A}$$

Performing the summation we find:

$$T^+ = N \cdot P(A) \cdot P(B|A, r_A) \cdot \frac{1 + P(B|A, r_A)(r_A - 1)}{r_A} \cdot \hat{\mu}_A \tag{7}$$

This equation can intuitively be understood as follows: the first factor gives the probability of choosing a type $A$ focal cell, the second the probability of choosing a type $B$ neighbor, and the third the probability that a type $A$ cell with at least one type $B$ neighbor reproduces. The term $1 + P(B|A, r_A)(r_A - 1)$ represents the expected number of $B$ neighbors, given that we know for sure that one neighbor is of type $B$ (the one that will be replaced by the offspring of $A$).

The rate $T^-$ can be found in a similar way, however we need to take into account that the replication neighborhood of type $B$ is smaller that its interaction neighborhood (i.e. $I_R \subseteq I_B$). The probability that the offspring of a $B$ cell replaces an $A$ neighbor is given by:

$$\frac{n_A}{r_B} \cdot \frac{P(A|B, r_A)}{P(A|B, r_B)}$$

the first factor is the probability of picking a type $A$ neighbor within the large neighborhood $I_B$, the second is the probability that an $A$ cell is part of the (small) replication neighborhood $I_R$, given that it is part of the (large) interaction neighborhood $I_B$. We thus find for $T^-$:

$$T^{-}=N\cdot\sum_{n_A=0}^{r_B}(1-P(A))\cdot P(B|B,r_B)^{r_B-n_A}\cdot P(A|B,r_B)^{n_A}\cdot\binom{r_B}{n_A}\cdot\frac{n_A}{r_B}\cdot\hat{\mu}_B\cdot\frac{n_A}{r_B}\cdot\frac{P(A|B,r_A)}{P(A|B,r_B)}$$

which sums to:

$$T^{-}=N\cdot(1-P(A))\cdot P(A|B,r_B)\cdot\frac{1+P(A|B,r_B)(r_B-1)}{r_B}\cdot\hat{\mu}_B\cdot\frac{P(A|B,r_A)}{P(A|B,r_B)} \tag{8}$$

When a $T^{+}$ or $T^{-}$ event happens the number of $X\leftarrow Y$ link changes by $\Delta^{+}_{XY}$ and $\Delta^{-}_{XY}$, respectively. We can use the conditional probabilities (Eq. 5) to calculate these quantities. Consider for example the case of a $T^{+}$ event, where an $A$ cell replaces a $B$ neighbor. The only links that change are the ones that start or end at the $B$ cell that will be replaced. To calculate the changes in links, we first have have to analyze the composition of the neighborhood of this $B$ cell. Using the conditional probabilities, we can calculate the expected number of $A$ and $B$ neighbors in both the small $S(B,r_A)$ and the large $S(B,r_B)$ neighborhood. We use our knowledge that the $B$ cell has at least one type $A$ neighbor (the cell that is about to reproduce) and find:

$$\begin{aligned}
n_A[S(B,r_A),T^{+}] &= 1+(r_A-1)P(A|B,r_A)\\
n_A[S(B,r_B),T^{+}] &= 1+(r_B-1)P(A|B,r_B)\\
n_B[S(B,r_A),T^{+}] &= (r_A-1)P(B|B,r_A)\\
n_B[S(B,r_B),T^{+}] &= (r_B-1)P(B|B,r_B)
\end{aligned}$$

here $n[S(B,r_A),T^{+}]$ indicates that this is the number of neighbors in the context of a small $S(B,r_A)$ neighborhood during a $T^{+}$ event.

These equations allow us to count the change of $B\leftarrow A$ links during a $T^{+}$ event. Before the replacement, all $B\leftarrow A$ links correspond to incoming links that start at an $A$ neighbor (within the large $S(B,r_B)$ neighborhood) that end at the central $B$ cell. We thus need to consider the number of $A$ neighbors within the large $S(B,r_B)$ neighborhood. In this neighborhood, we find $1+(r_B-1)P(A|B,r_B)$ $B\leftarrow A$ links before the replacement. After the replacement, $B\leftarrow A$ links corresponds to links that start at the newborn $A$ cell in the center and end at a $B$ neighbor (within the large $S(A,r_B)$ neighborhood). We thus need to consider the number of $B$ neighbors within the large $S(A,r_B)$ neighborhood. In this neighborhood, we find $(r_B-1)P(B|B,r_B)$ $B\leftarrow A$ links[1]after the replacement. Taking the difference between the number of links before and after the replacement we thus find that:

$$\Delta^{+}_{BA}=(r_B-1)P(B|B,r_B)-1-(r_B-1)P(A|B,r_B)$$

Next, let's consider $B \leftarrow B$ links. These links are defined on the large $S(B, r_B)$ neighborhood. The central $B$ cell has $(r_B - 1)P(B|B, r_B)$ $B$ neighbors and for each neighbor there are two $B \leftarrow B$ links: one incoming (pointing to the central cell) and one outgoing. Before the replacement, there were thus $2(r_B - 1)P(B|B, r_B)$ $B \leftarrow B$ links. After the replacement, there are no $B \leftarrow B$ links, because the central cell is now of type $A$. We thus find:

$$\Delta^+_{BB} = -2(r_B - 1)P(B|B, r_B)$$

Using similar reasoning, we can derive the changes in the other links for both the $T^+$ and $T^-$ events:

$$\begin{aligned}
\Delta^+_{AA} &= 2 + 2(r_A - 1)P(A|B, r_A) \\
\Delta^+_{AB} &= (r_A - 1)P(B|B, r_A) - 1 - (r_A - 1)P(A|B, r_A) \\
\Delta^+_{BA} &= (r_B - 1)P(B|B, r_B) - 1 - (r_B - 1)P(A|B, r_B) \\
\Delta^+_{BB} &= -2(r_B - 1)P(B|B, r_B) \\
\Delta^-_{AA} &= -2(r_A - 1)P(A|A, r_A) \\
\Delta^-_{AB} &= (r_A - 1)P(A|A, r_A) - 1 - (r_A - 1)P(B|A, r_A) \\
\Delta^-_{BA} &= (r_B - 1)P(A|A, r_B) - 1 - (r_B - 1)P(B|A, r_B) \\
\Delta^-_{BB} &= 2 + 2(r_B - 1)P(B|A, r_B)
\end{aligned} \tag{9}$$

where we have used the following neighborhood properties in the context of the $T^-$ event:

$$\begin{aligned}
n_A[S(A, r_A), T^-] &= (r_A - 1)P(A|A, r_A) \\
n_A[S(A, r_B), T^-] &= (r_B - 1)P(A|A, r_B) \\
n_B[S(A, r_A), T^-] &= 1 + (r_A - 1)P(B|A, r_A) \\
n_B[S(A, r_B), T^-] &= 1 + (r_B - 1)P(B|A, r_B)
\end{aligned}$$

We can then write the dynamical equation for $N_{X \leftarrow Y}$ as:

$$\frac{dN_{X \leftarrow Y}}{dt} = T^+ \cdot \Delta^+_{XY} + T^- \cdot \Delta^-_{XY} \tag{10}$$

We now have all the information to solve the dynamical equations 10: by inserting the rates as given in Eq. 7 and 8, the changes in links given by 9, and the conditional probabilities given by 5. The system of four coupled differential equations can be solved numerically, or we can

[1] Note that we use $P(B|B, r_B)$ even though after replication the cell at the center of the neighborhood is of type $A$. The reason is that the replication event only affects the central cell and not the wider neighborhood. Just before replication, a type $B$ cell was at the center of the neighborhood. The neighborhood is thus characterized by the conditional probabilities $P(Y|B, r)$ both directly before and directly after replication.

solve for the steady state analytically.

Before solving the dynamical equations, we change variables from $N_{X\leftarrow Y}$ to $P(A)$, $P(B|A, r_A)$, and $P(A|B, r_B)$:

$$P(A) = \tfrac{N_{A\leftarrow A}+N_{A\leftarrow B}}{rA\cdot N}, \quad P(B|A, r_A) = \tfrac{N_{A\leftarrow B}}{N_{A\leftarrow B}+N_{A\leftarrow A}}, \quad P(A|B, r_B) = \tfrac{N_{B\leftarrow A}}{N_{B\leftarrow A}+N_{B\leftarrow B}} \tag{11}$$

These new variables have direct biological meaning: the first corresponds to the global frequency of type $A$, the second corresponds to the local frequency of type $B$ in the interaction neighborhood of type $A$, and the last corresponds to the local frequency of type $A$ in the interaction neighborhood of type $B$.

Using Eq. 10 we can then write the dynamical equations for these variables:

$$\frac{dP(A)}{dt} = \frac{T^+\left(\Delta^+_{AA} + \Delta^+_{AB}\right) + T^-\left(\Delta^-_{AA} + \Delta^-_{AB}\right)}{rA} \tag{12}$$

$$\frac{dP(B|A, r_A)}{dt} = -\frac{P(B|A, r_A)\Big(T^-\left(\Delta^-_{AA} + \Delta^-_{AB}\right) + T^+\left(\Delta^+_{AA} + \Delta^+_{AB}\right)\Big) - T^-\Delta^-_{AB} - T^+\Delta^+_{AB}}{r_A \cdot P(A)} \tag{13}$$

$$\frac{dP(A|B, r_B)}{dt} = -\frac{P(A|B, r_B)\Big(T^-\left(\Delta^-_{BA} + \Delta^-_{BB}\right) + T^+\left(\Delta^+_{BA} + \Delta^+_{BB}\right)\Big) - T^-\Delta^-_{BA} - T^+\Delta^+_{BA}}{r_B \cdot (1 - P(A))} \tag{14}$$

Where $T^+$ is given by Eq. 7, $T^-$ by Eq. 8, $\Delta^+_{XY}$ and $\Delta^-_{XY}$ by Eq. 9, and where the conditional probabilities (Eq. 5) can be expressed in terms of our new variables as:

$$\begin{aligned}
P(A|A, r_A) &= 1 - P(B|A, r_A), & P(A|A, r_B) &= 1 - \frac{1 - P(A)}{P(A)} \cdot P(A|B, r_B) \\
P(B|A, r_A) &= P(B|A, r_A), & P(B|A, r_B) &= \frac{1 - P(A)}{P(A)} \cdot P(A|B, r_B) \\
P(A|B, r_A) &= \frac{P(A)}{1 - P(A)} \cdot P(B|A, r_A), & P(A|B, r_B) &= P(A|B, r_B) \\
P(B|B, r_A) &= 1 - \frac{P(A)}{1 - P(A)} \cdot P(B|A, r_A), & P(B|B, r_B) &= 1 - P(A|B, r_B)
\end{aligned} \tag{15}$$

We can solve the temporal dynamics of these equation numerically.

### 1.3.2 Steady state properties of cross-feeding communities

The equilibrium state can be found by setting Eq. 12-14 to zero and solving for $P(A)$, $P(B|A, r_A)$, and $P(A|B, r_B)$. We then find:

$$P(A) = \frac{\hat{\mu}_A \cdot \frac{r_A - 2}{r_A} + \left(\frac{\hat{\mu}_A}{r_A} - \frac{\hat{\mu}_B}{r_B}\right)}{\hat{\mu}_A \cdot \frac{r_A - 2}{r_A} + \hat{\mu}_B \cdot \frac{r_B - 2}{r_B}} \tag{16}$$

$$P(B|A, r_A) = \frac{r_A - 2}{r_A - 1} \cdot (1 - P(A)) \tag{17}$$

$$P(A|B, r_B) = \frac{r_B - 2}{r_B - 1} \cdot P(A) \tag{18}$$

$P(A)$ is the fraction of type A cells (i.e. it is a probability) and it thus has to satisfy $0 < P(A) < 1$. However, Eq. 16 can predict values for $P(A)$ which exceed these bounds. When Eq. 16 predicts values below 0 or above one this means that the two types cannot stably coexists.

In contrast, in a well mixed system, the growth rate of a cell only depends on the global frequency of the other cell type, from Eq. 6 it thus follows that:

$$\mu_A(P(A)) = (1 - P(A))\hat{\mu}_A, \qquad \mu_B(P(A)) = P(A)\hat{\mu}_B \tag{19}$$

At equilibrium the growth rate of both cell types is equal, which yields:

$$P(A)_{WM} = \frac{\hat{\mu}_A}{\hat{\mu}_A + \hat{\mu}_B} \tag{20}$$

## 1.4 Arbitrary communities of two interacting cell types

We can generalize our findings to any system of two cell types that interact with a defined number of neighboring cells. When the growth function is linear we can find an analytical solution for the steady state properties of the community; for non-linear growth functions we can solve the dynamics numerically.

### 1.4.1 Linear growth function

We consider the most general form of a linear growth function:

$$\begin{aligned} \mu_A(n_A, n_B) &= a_1 + a_2 \cdot n_A + a_3 \cdot n_B \\ \mu_B(n_A, n_B) &= b_1 + b_2 \cdot n_B + b_3 \cdot n_A \end{aligned} \tag{21}$$

here $a_1$ and $b_1$ represent the growth rate of type $A$ and $B$ cell when they are alone, $a_2$ and $b_2$ represent the increase in growth rate of type $A$ and $B$ cell when they are completely surrounded by their own type, and $a_3$ and $b_3$ represent the increase in growth rate of type $A$ and $B$ cell when they are completely surrounded by the other type. Our previous growth function (Eq. 6) can be recovered by setting $a_1, a_2, b_1, b_2 = 0$, $a_3 = \frac{\hat{\mu}_A}{r_A}$, and $b_3 = \frac{\hat{\mu}_B}{r_B}$. Because we assumed a birth-death process, it is important that the growth functions are positive for all possible neighborhood

compositions.

For this growth function, the rates $T^+$ and $T^-$ are given by:

$$T^+ = N \cdot P(A) \cdot P(B|A, r_A) \cdot \quad (22)$$
$$a_1 + a_2 \cdot P(A|A, r_A)(r_A - 1) + a_3 \cdot (1 + P(B|A, r_A)(r_A - 1))$$
$$T^- = N \cdot (1 - P(A)) \cdot P(A|B, r_B) \cdot \quad (23)$$
$$(b_1 + b_2 \cdot P(B|B, r_B)(r_B - 1) + b_3 \cdot (1 + P(A|B, rB)(r_B - 1))) \cdot \frac{P(A|B, r_A)}{P(A|B, r_B)}$$

Solving Eq 12 for steady state, using the new rates for $T^+$ (Eq. 22) and $T^-$ (Eq. 22), we find:

$$P(A) = \frac{a_3(r_A - 2) - b_2(r_B - 2) + (a_1 + a_2 + a_3) - (b_1 + b_2 + b_3)}{(a_3 - a_2)(r_A - 2) + (b_3 - b_2)(r_B - 2)} \quad (24)$$

As long as $0 < P(A) < 1$, the community has an equilibrium state where both types can coexist. We can also solve Eq. 13 and 14 at steady state and find that:

$$P(B|A, r_A) = \frac{r_A - 2}{r_A - 1} \cdot (1 - P(A)) \quad (25)$$
$$P(A|B, r_B) = \frac{r_B - 2}{r_B - 1} \cdot P(A) \quad (26)$$

The amount of clustering is thus the same, no matter what (linear) growth function is used.

### 1.4.2 Non-linear growth functions

When the growth function is non-linear, in general it is not possible to find an analytical solution for the steady state. However, the ODE system given by Eq. 12-14 can always be solved numerically. For general, non-linear, growth functions $\mu_A(n_A, n_B)$ and $\mu_B(n_A, n_B)$ the following rates for $T^+$ and $T^-$ apply (it is in general not possible to solve these sums analytically):

$$T^+ = N \cdot \sum_{n_B=0}^{r_A} P(A) \cdot P(A|A, r_A)^{r_A - n_B} \cdot P(B|A, r_A)^{n_B} \cdot \binom{r_A}{n_B} \cdot \mu_A(n_A, n_B) \cdot \frac{n_B}{r_A} \quad (27)$$

$$T^- = N \cdot \sum_{n_A=0}^{r_B} (1 - P(A)) \cdot P(B|B, r_B)^{r_B - n_A} \cdot P(A|B, r_B)^{n_A} \cdot \binom{r_B}{n_A} \cdot \mu_B(n_A, n_B) \cdot \frac{n_A}{r_B} \cdot \frac{P(A|B, r_A)}{P(A|B, r_B)} \quad (28)$$

Our model can also be extended beyond a birth-death process, Eq. 12-13 would still hold, as long as appropriate rate functions are used for $T^+$ and $T^-$. The conditional probabilities as

given by Eq. 15 and the expression for the changes in the number of links as given in 9 are independent of the replacement process and would thus also hold for e.g. a death-birth process.

# 2 Model application

## 2.1 Cross-feeding interaction

Here we will consider how local interactions can decrease the productivity of cross-feeding communities. In a spatial system the growth rate of cells depends on the average local frequency of the partner type. The average community growth rate is thus given by:

$$\langle \mu \rangle = P(A) \cdot P(B|A, r_A) \cdot \hat{\mu}_A + (1 - P(A)) \cdot P(A|B, r_B) \cdot \hat{\mu}_B \tag{29}$$

This equation follows from the fact that a fraction of $P(A)$ cells is of type $A$, on average each of these cells as $P(B|A, r_A)r_A$ neighbors of type $B$, and thus grows at an average rate (see Eq. 6) of $P(B|A, r_A)\hat{\mu}_A$. Similarly, a fraction of $1 - P(A)$ cells is of type $B$, on average each of these cells as $P(A|B, r_B)r_B$ neighbors of type $A$, and thus grows at an average rate of $P(A|B, r_B)\hat{\mu}_B$.

In well mixed system the growth rate of cells depends on the global frequency of the partner type. The average community growth rate is thus given by:

$$\langle \mu \rangle = P(A) \cdot (1 - P(A)) \cdot \hat{\mu}_A + (1 - P(A)) \cdot P(A) \cdot \hat{\mu}_B \tag{30}$$

This equation follows from the fact that a fraction of $P(A)$ cells is of type $A$, on average each of these cells as $(1 - P(A))r_A$ neighbors of type $B$, and thus grows at an average rate (see Eq. 6) of $(1 - P(A))\hat{\mu}_A$. Similarly, a fraction of $1 - P(A)$ cells is of type B, on average each of these cells as $P(A)r_B$ neighbors of type $A$, and thus grows at an average rate of $P(A)\hat{\mu}_B$.

## 2.2 Density dependent interaction

For some systems, we expect that growth is density and not frequency dependent. In this section we thus assume that growth rates increase linearly with the number, rather than the frequency, of neighbors of the other type:

$$\begin{aligned} \mu_A(n_A, n_B) &= \hat{\mu}_A \cdot n_B \\ \mu_B(n_A, n_B) &= \hat{\mu}_B \cdot n_A \end{aligned} \tag{31}$$

i.e. we use $a_1 = b_1 = a_2 = b_2 = 0$, $a_3 = \hat{\mu}_A$ and $b_3 = \hat{\mu}_B$. This linear growth function approximates well a more realistic Monod growth function, when the concentrations of the metabolite limiting growth are low in the environment, i.e. when they are below the saturation constant. Using Eq. 24, the steady state frequency is given by:

$$P(A) = \frac{\hat{\mu}_A(r_A - 2) + (\hat{\mu}_A - \hat{\mu}_B)}{\hat{\mu}_A(r_A - 2) + \hat{\mu}_B(r_B - 2)} \tag{32}$$

When $r_A, r_B \gg 1$ and $r_A \gg \frac{\hat{\mu}_B}{\hat{\mu}_A}$, this simplifies to:

$$P(A) = \frac{\hat{\mu}_A r_A}{\hat{\mu}_A r_A + \hat{\mu}_B r_B}$$

We thus see that the equilibrium frequency depends both on the strength of the interaction (given by $\hat{\mu}_A$ and $\hat{\mu}_B$) and the range of the interaction ($r_A$ and $r_B$); the type with the highest product $\hat{\mu} r$ will dominate the system.

## 2.3 Growth inhibition

Our model can also be used to model communities with antagonistic interactions, where cells inhibits each others growth (e.g. by producing bacteriocins or antibiotics). We assume that the growth rate of each cell decreases linearly with the frequency of partner cell that produce a toxic substance, from a basal growth rate $\mu_0$ that is the same for both types:

$$\begin{aligned} \mu_A(n_A, n_B) &= \mu_0 - \delta_A \cdot \frac{n_B}{r_A} \\ \mu_B(n_A, n_B) &= \mu_0 - \delta_B \cdot \frac{n_A}{r_B} \end{aligned} \tag{33}$$

i.e. we use $a_1 = b_1 = \mu_0$, $a_2 = b_2 = 0$, $a_3 = -\frac{\delta_A}{r_A}$ and $b_3 = -\frac{\delta_B}{r_B}$. As we assume a birth-death process, it is essential that growth rates remain positive for all possible neighborhood compositions. We thus assume that $\delta_A, \delta_B < \mu_0$.

Using Eq. 24, the steady state frequency is given by:

$$P(A) = \frac{\delta_A \cdot \frac{r_A - 2}{r_A} + \left(\frac{\delta_A}{r_A} - \frac{\delta_B}{r_B}\right)}{\delta_A \cdot \frac{r_A - 2}{r_A} + \delta_B \cdot \frac{r_B - 2}{r_B}} \tag{34}$$

In the case of growth inhibition, the community as a whole grows better in space than in an equivalent well-mixed system. This can be seen from Eq. 25 and 25: in a spatial system the local frequency of the other cell type is lower than its global frequency. Because cells grow faster when there are fewer cells of the other type, cells in a spatial system grow faster. Unlike in the case for cross-feeding, where cells need the other type to grow well and thus grow worse when they interact with few neighbors, with growth inhibition cells actually grow better when they are surrounded by their own type and thus grow better when they interact with few neighbors.

### 2.4 Application to an experimental cross-feeding community

We compared the prediction form our pair-approximation framework with data we previously obtained for an experimental synthetic cross-feeding community [1]. In S1 Table, we list all literature and measured parameter values used in this work. In S2 Table, we compare the parameterization from experimental data with the parameterization from the key biophysical parameters. In S3 Table, we compare the model predictions for the two parameterizations.

We previously found that the maximum growth rate predicted by the biophysical model (Eq. 6 in main text) tends to be larger than the measured maximum growth rate (see S2 Table and ref. [1]). This is primarily because we assumed that all metabolites can be used for growth (i.e. we assume Monod kinetics), while in reality metabolites are also needed for cell maintenance. This maintenance reduces the amount of metabolites available for growth and thus reduces $\hat{\mu}$ (see [1] for more details). However, this equation can accurately predict the ratio of the maximum growth rates between the two cell types (see S2 Table). From Eq. 16 it can be seen that our model only depends on the ratio of the maximum growth rates and not on their separate values, it is thus not needed to consider a more realistic growth function that includes maintenance costs.

## 3 References

1. Dal Co, A., van Vliet, S., Kiviet, D.J., Schlegel, S. & Ackermann, M. Short-range interactions govern the dynamics and functions of microbial communities. *Nat Ecol Evol* **4**, 366–375 (2020).
