# Supplementary material for: Global dynamics of microbial communities emerge from local interaction rules
Source: PLoS Comput Biol. 2022 Mar 4;18(3):e1009877. doi: 10.1371/journal.pcbi.1009877 (PMC8926250; doi:10.1371/journal.pcbi.1009877)
Supplement: S3 Text — Fig A: Testing assumption regarding replication neighborhood with simulations. Fig B: Validating pair-approximation with cellular automaton simulations. Fig C: Relation between patch size, interaction range, and spatial clustering. (PDF) [file pcbi.1009877.s003.pdf]

S3 Text

Supplementary Discussion

Contents

1 Replication Neighborhood **1**

2 Validating pair-approximation with cellular automaton simulations **3**

3 Relation between patch size, interaction range, and spatial clustering **3**

4 Spatial dynamics of cross-feeding communities **5**

5 References **6**

1 Replication Neighborhood

We modeled cell division using a ”birth-death” process, were cells that divide replace one of their neighbors. In real systems, neighboring cells are not replaced, but pushed away. However, we believe that a birth-death process can model real systems because it retains two fundamental properties of these real systems. First, the reproduction rate of individuals is proportional to their growth rate; second, the offspring of an individual is placed close by in space.

The main difference is that in the model a random neighboring cell is removed to make place for the new cell, while in reality a random neighboring cell is pushed away. This generates a motion that is transmitted through the community, and as a result of this, another cell is pushed out to the edge. Cells at the edge are likely to be lost, for example because of shear stress of fluids flowing around the community (as is the case in our experimental growth chambers). The result of pushing a neighbor away is thus similar to the removal of a random cell from the system: in both cases a random cell is removed from the system, though this happens locally in the model and globally in reality.

Both in the model and in reality cell types are clustered in space i.e. they form patches. Removing a cell from the local neighborhood of a dividing cell is thus not exactly equivalent to removing a random cell from the system; the local neighbor is more likely to be of the same type as the dividing cell compared to a random cell. This could generate a discrepancy between the model and the real system.

We further assumed that the replication neighborhood is identical to the smaller interaction neighborhood ($r_R = r_A$). This assumption was needed for mathematical tractability, however we can easily relax it in our (individual based) cellular automaton simulations. We thus ran a batch of simulations were we set the replication neighborhood to be identical to the smallest interaction neighborhood ($r_R = r_A$) and a batch of simulations were we keep the replication neighborhood fixed at Moore neighborhood ($r_R = 8$). The results of these two batches closely match, even for relatively large interaction neighborhoods ($r_A = 120$, Fig A), indicating that our simplifying assumption ($r_R = r_A$) does not affect our conclusions in any major way.

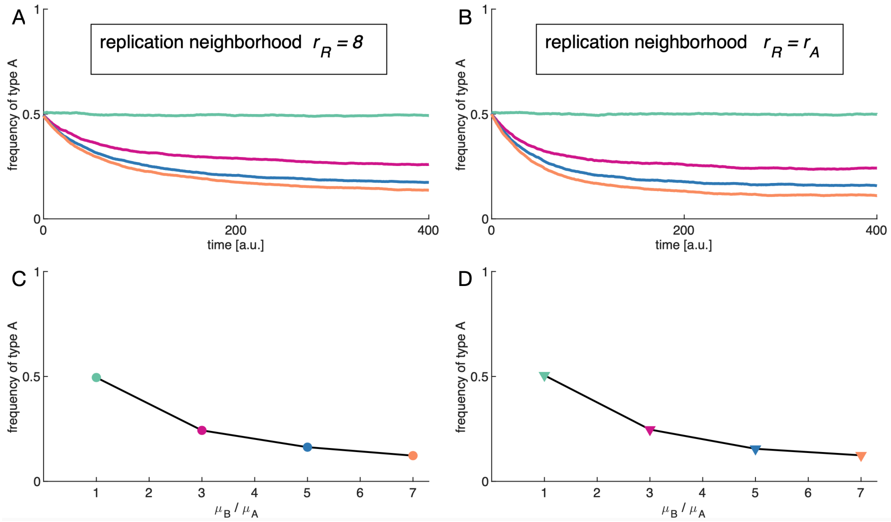

Fig A. Results of simulations are similar for a cellular automaton with replication neighborhood equal to the smallest interaction neighborhood ($r_R = r_A$) and for a cellular automaton with replication neighborhood equal to the Moore neighborhood ($r_R = 8$). (A) The fraction of type A is shown for four cellular automata simulations with $r_R = 8$ (Moore neighborhood). Each simulation is initialized with 50% of type A and 50% type B cells occupying a grid of 100x100. The four simulations have different values of μ_B/μ_A and have $r_B = r_A = 120$. (B) Four cellular automata simulations with replication neighborhood $r_R = r_A$ and all other parameters as in panel A. Note that the steady state fraction of type A is reached slightly slower in panel A then in panel B. The cellular automata with $r_R = 8$ (C) reaches the same equilibrium as the cellular automata with $r_R = r_A$ (D) when having the same μ_B/μ_A. Each circle (or triangle) indicates the steady state of one of the simulations in panel A (or B).

2 Validating pair-approximation with cellular automaton simulations

To test the validity of our pair-approximation results we compared its predictions to simulation results from a cellular automaton. We performed a scan of the two most relevant parameters of our model: the ratio of maximum growth rates and the ratio of interaction neighborhood sizes of

the two cell types. Overall the analytical predictions of the pair-approximation match well with the simulation results, however there are some differences for highly asymmetric communities (i.e. communities where the two cell types have different maximum growth rates, Fig B).

Specifically, pair-approximation and cellular automaton simulations are fully consistent in predicting which communities will collapse (gray areas in Fig B). Moreover, for communities in which the asymmetry in maximum growth rates is not too high, pair-approximation and cellular automaton quantitatively agree on both the global (i.e. equilibrium frequency, Fig B-A) and local properties (i.e. degree of clustering Fig B-B,C) of the community. However, for highly asymmetric communities the equilibrium frequency in simulations is more skewed (in favor of the majority cell type) than pair-approximation predicts. This difference is likely a consequence of stochastic fluctuation and/or of the finite population size in the simulations. Finally, for highly asymmetric communities the degree of clustering found in the simulations deviates from that predicted by pair-approximation, and this is most likely an artifact of how these quantities are calculated in the simulations. For highly asymmetric communities, there are only very few cells of the minority cell type and this makes the local frequency highly sensitive to stochastic fluctuations resulting in very noisy estimates of this quantity.

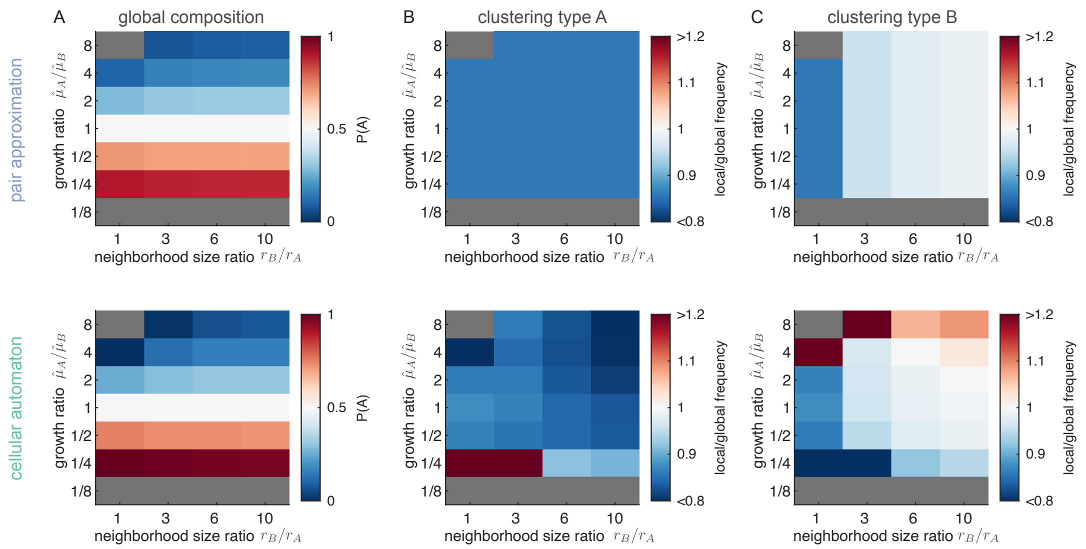

Fig B. Validating pair-approximation with cellular automaton simulations. Top row: analytical predictions of equilibrium properties of community as given by pair-approximation (Eq. 2 & 3 in main text); bottom row: equilibrium properties of communities simulated using a cellular automaton. (A): global composition of community ($P(A)$); (B-C): local composition of community (degree of clustering) for type A cells ($P(B|A, r_A)/P(B)$, panel (B)) and for type B cells ($P(A|B, r_B)/P(A)$, panel (C)). Gray areas indicate communities that collapse (i.e. where one of the two cell types goes extinct). Cellular automaton simulations were run on a randomly initialized grid of 100x100 cells with toroidal boundary conditions for 100'000 time steps. Equilibrium properties were calculated by averaging over the last 100 time points of the simulation. For each community, we show the average result of 9 simulations starting from different initial compositions ($P(A)$ equally spaced between 0.1 and 0.9). $r_A = 8$, $\hat{\mu}_A = 0.02$.

3 Relation between patch size, interaction range, and spatial clustering

We showed in the main text that cells in spatial systems tend to interact primarily with their own kind due to spatial clustering. As a result, for both cell types the local frequency of the partner type is lower than its global frequency (Eq. 3, Main text). Intuitively, this equation can be understood as follows: when the interaction range is short compared to the patch size, cell primarily interact with other cells within their own patch, and the local frequency of the partner type is much lower than its global frequency; however, when the interaction range is much larger than the patch size, cells interact with cells coming from many other patches and the local frequency of the partner type approaches its global frequency. To formalize this intuition, we here investigate the relation between patch size and the reduction in local partner frequency. We do this using the cellular automaton simulations, as it is not feasible to address this question within the pair-approximation framework.

Patch size can be calculated in various ways; here we use a simple heuristic approach: for each cell in the grid we move along both a vertical and an horizontal line and locate the closest points at which we find a cell of the other type. We consider these points to be the edges of the local patch as seen from the current focal cell and we calculate the width and height of the patch as the distance between these points. We do this procedure for all cells in the grid and estimate the patch size by averaging these values over all cells of a given type.

We then used simulations to evaluate how patch size and clustering depend on the size of the interaction neighborhood for a symmetric community ($r_A = r_B = r$ and $\hat{\mu}_A = \hat{\mu}_B$). We find that patch size increases with the size of the interaction neighborhood, but only weakly: a more than 20-fold increase in interaction neighborhood size results in less than a 2-fold increase in patch size (Fig C-A). As a result, the ratio between the interaction range (the radius of the extended Moore neighborhood) and the patch size quickly decreases as the neighborhood size is increased (Fig C-A). For the smallest interaction neighborhoods ($r = 8$) the interaction range is much smaller than the patch size (Fig C-B) and as a result the local partner frequency is strongly depressed (Fig C-B). However, as the interaction range is increased, it quickly becomes similar to the patch size and the local frequency of the partner type starts approaching the global frequency (Fig C-B,C).

4 Spatial dynamics of cross-feeding communities

To illustrate the spatial dynamics, we show four cellular automata simulations for four cross-feeding communities, covering different parts of the model parameter space explored in Fig B. The resulting dynamics are shown in S1 Video - S4 Video. For a movie of the experimental community, we refer the reader to Supplementary Movie 2 in reference [1]. All the cellular automata simulations are run on a 100x100 grid for 100'000 steps (or until community collapses)

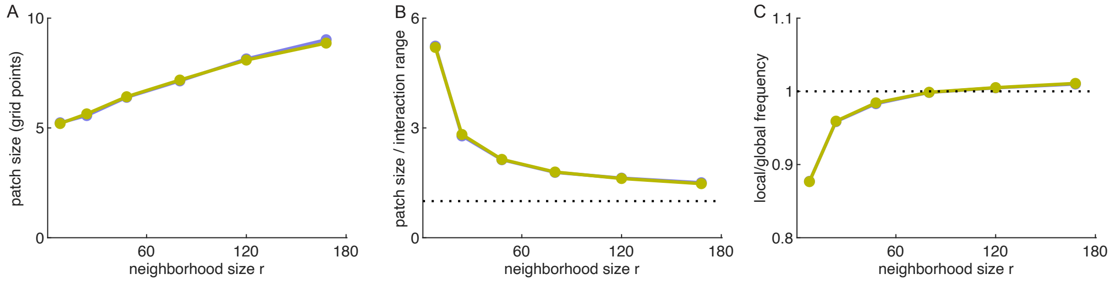

Fig C. Relation between patch size, interaction range, and spatial clustering. (A) Patch size (measured in grid units) as function of the size of the interaction neighborhood. Patch size increases weakly with the neighborhood size. (B) The ratio of the patch size to the interaction range as function of the size of the interaction neighborhood. As the neighborhood size is increases the interaction range quickly approaches the average patch size. (C) The degree of spatial clustering, i.e. the local over the global frequency of the partner type as function of the size of the interaction neighborhood. (A-C) Yellow curves correspond to cell type A, purple curves to cell type B. Cellular automaton simulations were run on a randomly initialized grid of 100x100 cells with toroidal boundary conditions for 100'000 time steps. Equilibrium properties were calculated by averaging over the last 100 time points of the simulation. For each community, we show the average result of 9 simulations started from different initial compositions ($P(A)$ equally spaced between 0.1 and 0.9). $\hat{\mu}_A = \hat{\mu}_B = 0.02$.

and the videos show one frame every 2'000.

5 References

1. Dal Co, A., van Vliet, S., Kiviet, D.J., Schlegel, S. & Ackermann, M. Short-range interactions govern the dynamics and functions of microbial communities. *Nat Ecol Evol* **4**, 366–375 (2020). https://doi.org/10.1038/s41559-019-1080-2
