# Supplementary material for: Global dynamics of microbial communities emerge from local interaction rules
Source: PLoS Comput Biol. 2022 Mar 4;18(3):e1009877. doi: 10.1371/journal.pcbi.1009877 (PMC8926250; doi:10.1371/journal.pcbi.1009877)
Supplement: S1 Table — (PDF) [file pcbi.1009877.s004.pdf]

S1 Table

Parameters values of biophysical model. All parameters of the model are taken from literature or measured. Values are show as mean ± standard error of the mean. l, w, and ρ were measured for 21 chambers, l, w were averaged over all cells within a given chamber before averaging over all chambers. ρ was estimated as the number of cells in the chamber divided by the total area occupied by cells. This area includes all intercellular free space, but excludes large empty areas in the chamber and was calculated by performing a morphological closing operation using a $1\mu m$ diameter on the segmented image. ^{a}: the concentration is normalized with the Monod constant of the growth curve.

Parameter	Description	Value	Source
$r^u_{\Delta P}$	uptake rate of proline	$2.04\ 1/s$	Literature [2]
$r^u_{\Delta T}$	uptake rate of tryptophan	$24.05\ 1/s$	Literature [3]
$D_{\Delta P}$	diffusion rate of proline	$8.79 \cdot 10^2\ \mu m^2/s$	Literature [4]
$D_{\Delta T}$	diffusion rate of tryptophan	$6.59 \cdot 10^2\ \mu m^2/s$	Literature [5]
$r^l_{\Delta P}$	leakage rate proline	$1.59 \cdot 10^{-5}\ 1/s$	Fitted [1]
$r^l_{\Delta T}$	leakage rate tryptophan	$6.04 \cdot 10^{-7}\ 1/s$	Fitted [1]
$\mathcal{I}^{\mathcal{C}}$	normalized concentration of metabolite in producing cell^a	20	Estimated [1]
μ^{wt}	growth on M9 media + 0.2% glucose	$1.29\ 1/h$	Measured [1]
ρ	volume density of cells	0.65	Measured [1]
ρ_{2D}	area density of cells	0.22 ± 0.01 cells/μm^2	Measured
l	average cell length	$5.2 \pm 0.1\ \mu m$	Measured
w	average cell width	$0.68 \pm 0.01\ \mu m$	Measured
β	conversion factor growth to interaction range	0.88	Measured [1]

References

1. Dal Co, A., van Vliet, S., Kiviet, D.J., Schlegel, S. & Ackermann, M. Short-range interactions govern the dynamics and functions of microbial communities. *Nat Ecol Evol* **4**, 366–375 (2020).
2. Grothe, S., Krogsrud, R. L., McClellan, D. J., Milner, J. L. & Wood, J. M. Proline Transport and Osmotic Stress Response in Escherichia coli K-12. *Journal of Bacteriology* **166**, 253-259 (1986).
3. Piperno, J. R., Oxender, D. L. Amino Acid Transport Systems in Escherichia coli. *Journal. Biol. Chem.* **243**, 5914-5920 (1968).

4. Wu, Y., Ma, P., Liu, Y. & Li, S. Diffusion coefficients of l-proline, l-threonine and l-arginine in aqueous solutions at 25°C. *Fluid Phase Equilibria* **186**, 27-38 (2001).
5. Longsworth, L. G. Diffusion Measurements, at 25°C, of Aqueous Solutions of Amino Acids, Peptides and Sugars. *Contrib. from Lab. Rockefeller Inst. Med. Res.* Nov. **20**, (1953).
