# Supplementary material for: Global dynamics of microbial communities emerge from local interaction rules
Source: PLoS Comput Biol. 2022 Mar 4;18(3):e1009877. doi: 10.1371/journal.pcbi.1009877 (PMC8926250; doi:10.1371/journal.pcbi.1009877)
Supplement: S2 Table — (PDF) [file pcbi.1009877.s005.pdf]

S2 Table

Parameter values of local interactions rules of experimental cross-feeding comminity. Comparison of model parameters for the parameterization based on the biophysical model and the one based on experimental data. The biophysical model overestimates the maximum growth rates, however it can accurately predict the ratio of the maximum growth rates, and this is the only factor that matters for the steady-state properties of the community. For the cellular automaton simulations the interaction neighborhood was set to an extended Moore neighborhood with radius d such that the number of cells in the Moore neighborhood $(2 \cdot d + 1)^2 - 1$ most closely matched the estimated number of neighbors r (1 and 5 grid units correspond to a neighborhood size of 8 and 120 cells, respectively). Intervals between brackets are 95% confidence intervals.

**Parameter**	**Description**	**Estimate from biophysical rates**	**Estimate from data**	**Units**
$R_{\Delta P}$	interaction range of ΔP	12.2	12.1 $(10.9, 13.3)$	μm
$R_{\Delta T}$	interaction range of ΔT	2.5	3.2 $(2.4, 4.1)$	μm
$r_{\Delta P}$	number of neighbors of ΔP	130	129 $(108, 150)$	cells
$r_{\Delta T}$	number of neighbors of ΔT	10	15 $(10, 20)$	cells
$d_{\Delta P}$	radius of extended Moore neighborhood of ΔP	5	5	grid units
$d_{\Delta T}$	radius of extended Moore neighborhood of ΔT	1	1	grid units
$\hat{\mu}_{\Delta P}$	maximum growth rate of ΔP	0.77	0.52 $(0.50, 0.55)$	$1/h$
$\hat{\mu}_{\Delta T}$	maximum growth rate of ΔT	0.22	0.15 $(0.14, 0.16)$	$1/h$
$\frac{\hat{\mu}_{\Delta P}}{\hat{\mu}_{\Delta T}}$	growth ratio	3.6	3.5 $(3.2, 3.8)$	
