# Supplementary material for: Global dynamics of microbial communities emerge from local interaction rules
Source: PLoS Comput Biol. 2022 Mar 4;18(3):e1009877. doi: 10.1371/journal.pcbi.1009877 (PMC8926250; doi:10.1371/journal.pcbi.1009877)
Supplement: S3 Table — (PDF) [file pcbi.1009877.s006.pdf]

S3 Table

Robustness of predictions to choice of model parameterization. Comparison of the predictions from the spatial (i.e. pair-approximation) model parameterized from the biophysical model, the spatial model parameterized from experimental data, and a well-mixed model, with experimental observations. Intervals between brackets are 95% confidence intervals. †: p-Values indicate result of Z-test between prediction of the Spatial model (with measured parameters) and of the Well-mixed model. ‡: p-Values indicate result of Z-test between prediction of the Spatial model (with measured parameters) and of the measured values.

**Variable**	**Spatial model (biophysical rates)**	**Spatial model (measured parameters)**	**Well-mixed model**†	**Measured value**‡
$P(\Delta T)$	0.20	0.21 $(0.19, 0.23)$	0.22 $(0.21, 0.24)$, $p = 0.3$	0.19 $(0.17, 0.20)$, $p = 0.04$
$\frac{P(\Delta T \mid \Delta P, r_{\Delta P})}{P(\Delta T)}$	0.99	0.99 $(0.99, 0.99)$	1, $p \ll 0.001$	0.92 $(0.84, 0.99)$, $p = 0.04$
$\frac{P(\Delta P \mid \Delta T, r_{\Delta T})}{P(\Delta P)}$	0.89	0.93 $(0.90, 0.95)$	1, $p \ll 0.001$	0.85 $(0.82, 0.88)$, $p \ll 0.001$
Community productivity relative to well-mixed	0.92	0.94 $(0.92, 0.95)$	1, $p = 0.3$	0.87 $(0.85, 0.90)$, $p = 0.35$
