# Supplementary material for: Global dynamics of microbial communities emerge from local interaction rules
Source: PLoS Comput Biol. 2022 Mar 4;18(3):e1009877. doi: 10.1371/journal.pcbi.1009877 (PMC8926250; doi:10.1371/journal.pcbi.1009877)
Supplement: Attachment — Submitted filename: report_van_vliet_2021.pdf [file pcbi.1009877.s011.pdf]

The manuscript aims at a quantitative description of the population-level properties of a simple community composed of two cell types, which exchange metabolites necessary for growth. Such mutualistic communities have been characterized theoretically and experimentally mostly in well-mixed environments, where their cross-feeding allows different cell types to coexist. Building on their previously developed models that describe population dynamics based on estimates of the relevant interaction spatial scales, the authors bring the following novel contributions:

1. They extend the previous approach, developed for describing growth in two spatial dimensions, to include a third dimension. In this way, effective interaction ranges can be connected directly to cell-level traits.
2. They propose a pair-approximation method to derive the frequency of the two types based on maximal growth rates and interaction radii. Methods of this kind are already available (see for instance Ellner, Pair Approximation for Lattice Models with Multiple Interaction Scales, JTB 2001), but the previous parametrization allows the authors to apply this specifically to the studied experimental system.
3. They compare the steady-state solution of the model to that measured in a bacterial two-strains community observed in a microfluidic reactor.

The topic of how mutualistic communities structure in space and time is certainly very important and has witnessed a recent increase in attention, in particular in relation to observation of natural communities.
Mechanistic approaches like those developed by the authors complement this kind of studies by linking population-level properties to cell-level traits that are measurable and – in some cases – controllable in the lab.
Overall, the manuscript is clearly written and enjoyable to read.
I have a few concerns about the novelty and impact, whose magnitude seem to me a bit overstated.
The mathematical developments presented are novel and interesting, though they could have been more thoroughly related to similar previous approaches.
The pair-approximation method proposed here is general for describing binary communities that differ in reproduction rate and on the scale of spatial interactions. Its characterization could in my opinion be pushed further in order to clarify the link with spatial patterns and their dynamics.
Moreover, I found the comparison with the data a bit superficial, especially considering the claim that the mathematical method proposed provides predictive tools that can be applied to more general communities. I feel that either the comparison is expanded, or the accent should be put on the theoretical advances, that are interesting in themselves. As it is, the message does not come through very convincingly.

I provide below more details on aspects I found could be improved.

A. Some key quantities that are central for the interpretation of the model should be more clearly defined. For instance, the relation between (space-averaged) local and global frequency could be put in relation to the asymptotic pattern, after coarsening reduces cell turnover to the interfaces.

As the paragraph starting on line 232 is formulated now, it sounds as if both local frequencies can be simultaneously smaller than their well-mixed value, which is confusing (but maybe it is due to a missing part in the sentence on line 235). Figure 3 and its caption are correspondingly unclear. The fact that global (? panel A) frequency can be 0 or 1 if both types are needed for survival, is counterintuitive in a population of constant size. Growth is a similarly unclear term. Usually, growth is the net result of reproduction and death. As in this model cells do not die, it coincides with reproduction, consistent with the measure of elongation rate in the experiments. I think it would be much clearer if it was stated so. Indeed, zero growth of a population of constant size (as depicted in fig. 4) is more understandable as an absence of turnover. Growth of a microbial population is most often associated to a change in total cell number. This distinction is important when one talks about 'community growth', which should be explicitly defined in the main text. I think that showing a few snapshots of the simulations (and, if possible, of the experiments) would be very helpful in making clear what observables are represented by the model. Presenting an example of 'community collapse' would be as well useful.

B. In order for pair approximation to be applied, the only quantities that matter are 4 parameters that can be estimated either from cell-level traits, or measured directly at the population level. The authors do both, and they provide two alternative (2-D) and (3-D) estimates for the former. However, they do not discuss why should the 3-D formulation be more appropriate for their experimental system (indeed, in order to segment the time lapse movies, I guess growth in the reactors happens in 2-D). To me, it is not evident why the 3-D extension is needed.

C. I would have appreciated more discussion in the main text about the correspondence between the model simulations and the pair approximation. Since pair approximation relies on a series of assumptions, understanding in what limits the authors expect the approximation to break down would have been be very useful in order to evaluate their value for describing real systems. For instance, the comparison of the approximated equations with the simulations could go beyond the equilibrium composition – which in the experiment, moreover, is not that far from the well-mixed case. Based on the microscopic model, I guess the authors could estimate the time scale of the convergence to the equilibrium, whereas in figure 6 simulations are depicted in arbitrary units (therefore, it is not that surprising that the three dynamics are similar).

D. Experiments should be described in more detail. In the main text. First of all, how are the 22 examined communities different? Can confidence intervals be provided on the ranges estimated from the microscopic parameters, or at least those measured effectively? This would be important for the comparison with the model(s). A statistical analysis of the significance of pair approximation with respect to predictions for the well-mixed system would also seem appropriate. Indeed, the central result of the paper is that pair approximation can be used to gather

additional information with respect to what is obtained from simple mean-field equations. This is at present not obvious.
Ideally, in order to evaluate the predictive capabilities of the model, one would like the comparison to be carried out in (at least two) cases when microscopic parameter differ, so as to have an effect on the interaction ranges. Even if this is not feasible for this paper, it would be interesting to know what experiments could be foreseen in order to test the generality of the results as claimed in the manuscript.

Minor points:

i. Figure 4 is referred to as Fig. 5 in the paragraph starting on line 262
ii. Line 398-400: How do previous works compare with the predictions of the current work?
iii. Line 452: why should an individual's reproduction rate depend on the average reproduction rate of the population?
