# Supplementary material for: Global dynamics of microbial communities emerge from local interaction rules
Source: PLoS Comput Biol. 2022 Mar 4;18(3):e1009877. doi: 10.1371/journal.pcbi.1009877 (PMC8926250; doi:10.1371/journal.pcbi.1009877)
Supplement: Attachment — Submitted filename: Response_to_reviewers_PLOSCB.pdf [file pcbi.1009877.s012.pdf]

We would like to thank the editor and both reviewers for taking the time to evaluate our manuscript. We were pleased to read that both reviewers were overall positive about our work. We appreciate their constructive criticism which has allowed us to improve our manuscript. Specifically, we have made the following major changes:

- Complete rewrite of abstract to shorten and refocus it
- Rewrite of introduction to clarify how our current work is different from our previous work
- Rewrite of Model and Result sections to present our main results in a more organized and unified way
- Insertion of additional analysis of the cellular automaton simulations to compare it to pair approximation
- Addition of a detailed discussion of how our spatial model compares to a well-mixed model
- Addition of details on the experimental system and a statistical analysis between the data and predictions from spatial and non-spatial models
- Addition of supplementary movies to show dynamics of simulations

We discuss these changes in detail, as well as many additional smaller changes, in the point-by-point response below. Overall, we believe that we have been able to address all concerns raised by the reviewers and hope that you will accept the revised version of this manuscript for publication in *PLOS Computational Biology*.

Reviewer #1:

The manuscript aims at a quantitative description of the population-level properties of a simple community composed of two cell types, which exchange metabolites necessary for growth. Such mutualistic communities have been characterized theoretically and experimentally mostly in well-mixed environments, where their cross-feeding allows different cell types to coexist. Building on their previously developed models that describe population dynamics based on estimates of the relevant interaction spatial scales, the authors bring the following novel contributions:

They extend the previous approach, developed for describing growth in two spatial dimensions, to include a third dimension. In this way, effective interaction ranges can be connected directly to cell-level traits.

They propose a pair-approximation method to derive the frequency of the two types based on maximal growth rates and interaction radii. Methods of this kind are already available (see for instance Ellner, Pair Approximation for Lattice Models with Multiple Interaction Scales, JTB 2001), but the previous parametrization allows the authors to apply this specifically to the studied experimental system.

They compare the steady-state solution of the model to that measured in a bacterial two-strains community observed in a microfluidic reactor.

The topic of how mutualistic communities structure in space and time is certainly very important and has witnessed a recent increase in attention, in particular in relation to observation of natural communities. Mechanistic approaches like those developed by the authors complement this kind of studies by linking population-level properties to cell-level traits that are measurable and – in some cases – controllable in the lab.

Overall, the manuscript is clearly written and enjoyable to read.

I have a few concerns about the novelty and impact, whose magnitude seem to me a bit overstated.

The mathematical developments presented are novel and interesting, though they could have been more thoroughly related to similar previous approaches. The pair-approximation method proposed here is general for describing binary communities that differ in reproduction rate and on the scale of spatial interactions. Its characterization could in my opinion be pushed further in order to clarify the link with spatial patterns and their dynamics.

Moreover, I found the comparison with the data a bit superficial, especially considering the claim that the mathematical method proposed provides predictive tools that can be applied to more general communities. I feel that either the comparison is expanded, or the accent should be put on the

theoretical advances, that are interesting in themselves. As it is, the message does not come through very convincingly.

I provide below more details on aspects I found could be improved .

Response: We thank the reviewer for the time and effort invested in evaluating our work, for their constructive feedback, and for their overall positive evaluation of our work. We have made significant changes to our manuscript to address the reviewer's comments which we will describe in detail below. Most importantly, we followed the reviewer's suggestion above and now focus primarily on the theoretical aspects of our work and de-emphasized the empirical parts. Moreover, we have taken extra care to be extremely careful in how we interpret the relation between the experimental data and model predictions, making clear that follow up work is needed for a proper validation of our framework.

We also like to thank the reviewer for pointing out the work by Ellner, which we unfortunately neglected to cite before. Although this is indeed a relevant paper, which we now properly cite, we would like to point out that this work still differs significantly from our approach: Ellner considered the impact of multiple interactions that have different ranges and that impact different cellular processes (birth and death rate respectively), however, and crucially, the interactions were still assumed to be the same for both cell types. This assumption allows for the use of undirected graphs. In our case, however, we allow the different cell types to interact at different spatial ranges which requires the use of directed graphs. The change from undirected to directed graphs requires a new mathematical framework which we describe in the SI. To the best of our knowledge, such use of directed graphs is novel within the context we consider. We now discuss this point in the main text as follows (line 170):

Our use of directed graphs is new in the field: all previous models assumed that interactions were symmetric between cell types; some models assumed that interactions affecting birth and death rates of cells occurred at different ranges, but these ranges were the same for the different cell types [Ellner:2001].

Reviewer comment 1.1

A. Some key quantities that are central for the interpretation of the model should be more clearly defined. For instance, the relation between (space-averaged) local and global frequency could be put in relation to the asymptotic pattern, after coarsening reduces cell turnover to the interfaces. As the paragraph starting on line 232 is formulated now, it sounds as if both local frequencies can be simultaneously smaller than their well-mixed value, which is confusing (but maybe it is due to a missing part in the sentence on line 235).

Response: we apologies for the editing error that caused part of line 235 to drop-out, this has now been corrected. Although at first sight it might seem counterintuitive that both local frequencies are smaller than the well-mixed frequency, this is indeed what our model shows happens in spatial systems. We have added the following explanation to the main text which we hope can resolve the confusion (line 273):

Both cell types are affected by this reduction in the local frequency of the partner type. This can be understood as follows: in a spatial system, cells of both types place their offspring close by in space and as a result they form patches. Within each patch, the frequency of the partner type is (much) lower than the global frequency. When interaction ranges are short, cells on average interact mostly within their own patch, and thus mostly interact with their own type (Fig S3, see Supporting Information, section 4.3). The average cell thus interacts with fewer cells of the other cell type than it would in a well-mixed system. In other words: in spatial systems, the local frequency of the partner type is always lower than its global frequency (as is shown by Eq.3). The difference will grow smaller as the interaction range becomes large compared to the patch size (Fig S3).

In addition, we have added a section and figure to the SI (SI section 4.3, Fig S3) where we explore the relation between patch size, interaction range, and the ratio of local to global frequency, to further illustrate this point.

Reviewer comment 1.2

Figure 3 and its caption are correspondingly unclear. The fact that global (? panel A) frequency can be 0 or 1 if both types are needed for survival, is counterintuitive in a population of constant size.

Response: we have updated both figure and caption to address this point. In the figure we removed data points at a frequency of 0 and 1. In the caption we added the following clarification:

When either type becomes fixed (i.e., P(A)=0 or P(A)=1) cells can no longer reproduce. In natural communities, where populations sizes can change, this would lead to the collapse of the community; however, in our model we assume a constant population size, so in this case the community persists even though there is no longer any turnover of cells (i.e., community productivity drops to 0).

Reviewer comment 1.3

Growth is a similarly unclear term. Usually, growth is the net result of reproduction and death. As in this model cells do not die, it coincides with reproduction, consistent with the measure of elongation rate in the experiments. I think it would be much clearer if it was stated so. Indeed, zero growth of a population of constant size (as depicted in fig. 4) is more understandable as an absence of turnover. Growth of a microbial population is most often associated to a change in total cell number. This distinction is important when one talks about 'community growth', which should be explicitly defined in the main text.

Response: to avoid confusion we have changed our terminology, and now use the term "community productivity" which we define explicitly as the gross rate at which new biomass is produced. In addition, we have added the following discussion to the main text to explain how this community productivity relates to the population growth rate and turnover rate (line 296):

To quantify this effect, we calculate the average birth rate of cells in the community; this quantity corresponds to the gross rate at which new biomass is produced and we refer to it as the community productivity. It is important to note that the net change in biomass (i.e., the change in population size) also depends on the rate of cell death/loss. In our model we assume a constant population size, so the net rate of biomass production is always zero. Biologically, this corresponds to biofilms where cell growth and loss balance each other, e.g., because cells at the edge of the biofilm are flushed away. Even in such scenarios where population sizes are constant, community productivity is still an important quantity as it measures the turn-over rate of cells, which in turn affects many relevant quantities such as the overall metabolic rate in the community.

Reviewer comment 1.4

I think that showing a few snapshots of the simulations (and, if possible, of the experiments) would be very helpful in making clear what observables are represented by the model.

Response: we have added several SI movies (SI Video 1- 4) showing the dynamics of the simulations in different parts of the model parameters space. A movie of the experiments has been published as part of our previous work (Dal Co et al, Nat. Eco. Evo. 2020), we have added a link to this movie to the relevant SI section 4.4.

Reviewer comment 1.5

Presenting an example of 'community collapse' would be as well useful.

Response: we have added an example as SI Video 4.

Reviewer comment 1.6

B. In order for pair approximation to be applied, the only quantities that matter are 4 parameters that can be estimated either from cell-level traits, or measured directly at the population level. The authors do both, and they provide two alternative (2-D) and (3-D) estimates for the former. However, they do not discuss why should the 3-D formulation be more appropriate for their experimental system (indeed, in order to segment the time lapse movies, I guess growth in the reactors happens in 2-D). To me, it is not evident why the 3-D extension is needed.

Response: our experimental system is indeed quasi-2D and can thus be described using our previous 2D biophysical model. However, many natural communities are 3D and our goal here was to develop a modelling-framework that can generally be applied to all relevant (natural) communities and not just to our experimental system. In fact, a major criticism of our previous work is that it only applies to 2D systems and thus lacks relevance for natural communities. A primary motivation of our current work was to address this shortcoming by extending our previous results to 3D. Although we were unable to test

our model's predictions with data from 3D communities within the scope of the current manuscript, our expectation is that this will be possible in future work. For that reason, we believe that the 3D extension is essential for the future applicability of our modelling framework.

The reviewer's comment, however, made us realize that we did not properly explain our motivation for the 3D extension in our manuscript. We have thus rewritten the relevant parts of the introduction (line 94) and result (line 131) sections to explicitly discuss the above-mentioned points.

Reviewer comment 1.7

C. I would have appreciated more discussion in the main text about the correspondence between the model simulations and the pair approximation. Since pair approximation relies on a series of assumptions, understanding in what limits the authors expect the approximation to break down would have been be very useful in order to evaluate their value for describing real systems. For instance, the comparison of the approximated equations with the simulations could go beyond the equilibrium composition – which in the experiment, moreover, is not that far from the well-mixed case. Based on the microscopic model, I guess the authors could estimate the time scale of the convergence to the equilibrium, whereas in figure 6 simulations are depicted in arbitrary units (therefore, it is not that surprising that the three dynamics are similar).

Response: we have added several additional results to compare pair-approximation (PA) to the cellular automaton (CA) simulations. Specifically, we tested the two main assumptions we made in deriving PA: 1) the assumption that the replication neighborhood is identical to the smaller of the interaction neighborhoods, and 2) the central assumption that the effects of spatial structure can be captured by only tracking pair-wise frequencies. The first assumption we had already tested in the first version of the manuscript and we showed that it does not significantly affect our results (Supplementary Discussion section 4.1 and Fig. S2). We now added additional simulation results to test the second assumption. Specifically, we repeated the parameter space scan shown in Fig 5A (done with PA) using our CA simulations and compare the predictions for the global composition of the community and the degree of spatial structure. We found that overall the predictions of PA match well those of the CA simulations, unless communities are highly asymmetric (see new Fig S3). All other major assumptions (e.g., with regards to the functional shape of the local rules) were used both in deriving our PA results and in setting up the CA simulations, so simulations could provide no further insight here. Taken together, our CA simulation thus show that the assumptions required for PA do not greatly affects its predictions. We have added a discussion of these points to the Supplementary Information (Section 4.2), and briefly highlight this in the main text (line 195):

We implemented our model in two complementary ways: we used a cellular automaton to simulate the dynamics numerically (see Methods) and we used pair approximation to derive analytical predictions. Overall we find that both methods agree well, as long as the communities are not too asymmetric (i.e., dominated by one of the two cell types; see Fig S2 and Supporting Information, section 4.2).

Regarding analyzing the non-equilibrium dynamics of our model: our primary goal here was to derive the steady-state properties of the community; a full investigation of the non-equilibrium dynamics would require a great deal of additional work and would, in our opinion, require a dedicated treatment by itself. The reason for this is that, for example, it is non-trivial to convert iterations in the cellular automaton (one birth event per iteration) to proper clock times. Likewise, we previously used time-rescaling to derive the dynamical equations for PA. We realize that we did not make our focus on the steady state properties clear enough before, and we now explicitly point this out in all relevant parts of the manuscript (e.g., line 102).

Having said that, we did attempt to address the Reviewer's comments as far as we could. Specifically, we were able to re-derive the dynamical equations of PA without rescaling time. Using these equations, we can now compare the approximate time required to equilibrate between the data (Fig. 6A) and pair-approximation (Fig 6B). We added a brief comment on this to the main text (line 392):

We did not set out to accurately model the temporal dynamics of the community before it reaches steady-state; nonetheless both in the data and in pair-approximation we observed that this process takes on the order of tens of hours, though the observed dynamics are slower by about a factor 2 (Fig 6A-B). A more detailed investigation is needed to assess the extent to which pair-approximation can be used for non-equilibrium dynamics, but we leave this for future work.

Reviewer comment 1.8

D. Experiments should be described in more detail. In the main text. First of all, how are the 22 examined communities different?

Response: we have added the following description to the main text (line 370):

We followed the dynamics inside 22 replicate chambers using time-lapse microscopy and used an automated image analysis workflow to quantify the composition, spatial arrangement, and growth rate of the community over time. These chambers are identical to each other, except for the initial frequency of the two cell types, which was determined by chance during the stochastic cell-loading process (see reference [11] for details)

Reviewer comment 1.9

Can confidence intervals be provided on the ranges estimated from the microscopic parameters, or at least those measured effectively? This would be important for the comparison with the model(s).

Response: the parameters estimated from the biophysical rates, which we use in the main text, were obtained from the literature; unfortunately, most of the consulted papers did not provide confidence intervals for the biophysical parameters. For the parameters estimated from the data, however, we were able to obtain confidence intervals and we used these to also estimate the confidence intervals of the predicted community level properties. We have added these confidence intervals to Table S2 (for parameter values) and Table S3 (for model predictions).

Reviewer comment 1.10

A statistical analysis of the significance of pair approximation with respect to predictions for the well-mixed system would also seem appropriate. Indeed, the central result of the paper is that pair approximation can be used to gather additional information with respect to what is obtained from simple mean-field equations. This is at present not obvious.

Response: unfortunately, we lack confidence intervals for the biophysical rates which we use to parametrize our model in the main text (see above), as a result, a statistical analysis is not possible for the results presented in the main text (as both pair approximation and the well-mixed model only give a prediction for the central estimate). However, we agree with the reviewer that it is important to highlight the differences between the predictions of pair-approximation and the well-mixed model in more detail. We have thus added a detailed discussion of this point to all relevant places in the main text (e.g., see lines 251, 400). In addition, we have added the predictions of the well-mixed model to Table S3 and compare them statistically to the prediction by the pair approximation model parameterized from the data.

Reviewer comment 1.11

Ideally, in order to evaluate the predictive capabilities of the model, one would like the comparison to be carried out in (at least two) cases when microscopic parameter differ, so as to have an effect on the interaction ranges. Even if this is not feasible for this paper, it would be interesting to know what experiments could be foreseen in order to test the generality of the results as claimed in the manuscript.

Response: we absolutely agree with the reviewer that additional data points to test our model would be beneficial and we have previously attempted to obtain these by repeating our experiments with additional microbial cross-feeding communities with different microscopic parameters. However, these experiments are technically highly complex; due to resource limitations we unfortunately had to give up on this line of experiments. For similar reasons, it is not feasible, as already pointed out by the Reviewer, to perform these experiments as part of the current revision. Instead, we follow the Reviewer's suggestion and added the following paragraph to the discussion where we describe which experiments could be pursued in future work to further evaluate our model (line 483):

To properly validate our model, it would be essential to perform additional experiments with different communities. This could be done in several ways: biophysical parameters (and thus local rules) vary between molecules, species, and transport pathways; changing any of these would place a community in a different part of the model parameter space. For example, one could repeat our experiments using communities that exchange different building blocks or that are composed of different species.

Moreover, one could directly change relevant biophysical parameters using genetic engineering, for example by tuning the uptake rate of molecules by manipulating the corresponding uptake pathways. Finally, thanks to recent advances in image analysis techniques [37], it has now become feasible to perform similar experiments in three-dimensional biofilms, making it possible to experimentally test the effects of dimensionality on community-level properties.

Reviewer comment 1.12

Figure 4 is referred to as Fig. 5 in the paragraph starting on line 262

Response: this error has been corrected.

Reviewer comment 1.13

Line 398-400: How do previous works compare with the predictions of the current work?

Response: we have added the following lines to discuss the differences and similarities of the cited work to our own (line 458):

Our biophysical model suggests that the interaction range is indeed comparable between two- and three-dimensional systems (Fig S1, see Supporting information). However, a recent study came to the opposite conclusion: van Tatenhove-Pel et al. found that the interaction range in three dimensions is shorter than in two dimensions [21]. This difference can likely be explained by the different spatial arrangement that were considered: we calculated the interaction range around larger patches of producer cells, while van Tatenhove-Pel et al. calculated it for a single, isolated, producer cell. When there is only a single producer cells, only few molecules are produced and increasing dimensionality decreases the interaction range as they are spread over a larger volume. However, when the number of producer cells is larger (as will be the case in most communities that are not too asymmetric) this effect will become less important and the interaction range becomes largely independent of dimensionality, as we found. Despite these differences, both our work and that of van Tatenhove-Pel et al. show clearly that spatial structure and dimensionality have important effects on the dynamics of microbial communities.

Reviewer comment 1.14

Line 452: why should an individual's reproduction rate depend on the average reproduction rate of the population?

Response: in previous work, all growth rates were normalized by the average reproduction rate of the population in order to rescale time in units of the overall rate of population turn-over, and we previously followed this convention. However, we have now removed this factor as we no longer rescale time (this was done in other to address point 1.7 of the Reviewer).

Reviewer #2:

In previous work, the authors examined the growth of two co-dependent species (A and B) that each lack the ability to produce one essential amino acid that is produced by the partner specie. Experiments were conducted in 2D layers. They found that a cell could benefit from partner specie cells only if these where within a small interaction range set by the uptake rate of the molecule. They also found that the growth rate of a cell depended on the fraction of cells of the partner species within its interaction range and that maximum growth rate was set by the leakage rate of the exchanged amino acid.

In this paper the authors use a graph-base model to show that these local rules of interaction are sufficient to predict several global community measures such as community composition, spatial arrangement, and growth rate.

The main findings are:
- Developed a simple graph-based model that encapsulates local rules of interactions and measurements of microscopic parameters and that can predict community-level properties. Improvement on past graph-based models is that the interaction range can be different for different cells/metabolites, which the author show is necessary to understand the experimental community.

- Analytical expressions for the steady state global and local compositions of the community as a function of the local rules, including predictions for differences between 2D and 3D communities.
- Exploration of the effects of different neighborhood sizes, from very short range to well-mixed, and different growth rates, and prediction of the range of parameter values that leads to the collapse of the community.
- Validation with experimental measurements of microbial community.

These are all good results that will be useful for the field. Being able to make predictions that cross scales is rare and extremely valuable. However, in its current form the paper risks being ignored because it is poorly written and organized, which makes it extremely tedious to read.

Response: we thank the reviewer for the time and effort invested in evaluating our work, for their feedback, and for their overall positive evaluation of our work. We regret that the format we chose made our paper tedious to read for this reviewer. We have extensively rewritten the manuscript incorporating the Reviewer's suggestion, and hope that this will address these issues. We will highlight these changes in detail below.

Main Concerns:

Reviewer comment 2.1

The abstract very long and unfortunately vague. It fails to clearly spell out the key new finding as opposed to what was learned from the previous study. This is especially true for lines 25-30: "For these cross-feeding communities, the community growth rate is reduced when cells interact only with few neighbors; as a result, some communities can co-exist in a well-mixed system, where cells can interact with all other cells, but not in systems where cells can interact only with close by neighbors." What is new and what is past results in that statement? I recommend rewriting the abstract entirely, making sure to clearly spell out the key new findings.

Response: we have followed the Reviewer's suggestion and have completely rewritten the abstract. We have shortened it and shifted focus to the key new findings as very nicely summarized above by the Reviewer.

Reviewer comment 2.2 & 2.3

The artificial split between the Model section and the Result section forced me to go back and forth between the two section and got me lost several times. This is a Computational Biology paper. The model is one of the main contributions of the paper. It is tedious to go through the model first without discussion of the plots which provide meaning to the equations. I recommend reorganizing so that derivations of key equations and plots and interpretation are together. For example: the sections "Steady state community properties" and "Local cell-cell interactions predict community properties" and Fig 3 clearly belong together.

This lack of organization reflects the poor overall organization of the paper. In its current form it is difficult for the reader to follow the logic of question – model – plot – interpretation, next question – model – plot – interpretation, and so on… instead the same question/concept is treated in various places. For example, the results on community collapse are all over the places: could all text/figures/equations related to the analysis of collapse in cross feeding communities be in one place?

Response: we have completely rewritten the relevant parts of the Model and Result sections. Specifically, we have merged the "Steady state community properties", "Local cell-cell interactions predict community properties", and "Small interaction neighborhoods can lead to collapse of cross-feeding communities". These are now combined in the single section "Steady state community properties" which has been moved to the beginning of the Result section (line 229). This section has been completely rewritten and re-organized, and we now discuss the relevant equations, the corresponding figures, and their interpretation in the same section. In particular, the results regarding community collapse have been fully integrated in this section and directly follow our derivation of the community growth rate (now called "community productivity" in response to a comment from Reviewer 1).

Although we agree with the reviewer that this is primarily a Computational Biology paper, we believe that it is also of interest to empirical researchers who might want to apply our framework to their model communities. For that reason, we consciously decided to split the technical details of our model from its use and application to keep the manuscript also accessible for less mathematically/computationally inclined readers. Although this format worked well for some readers (see the positive comments of Reviewer 1 regarding our writing), we now realize that for other readers our choice did not work out well. We believe that with the rewrites discussed above we have been able to find a good compromise that will work well for all readers; we have kept a separate model section where we discuss the more technical aspects of our model, but have followed Reviewer 2's suggestions and merged the presentation of our main equations (Eq. 1&3 describing the steady state properties of the community), with the corresponding figures and interpretation, all of which is now presented at the start of the Result section.

Minor Comments:

Reviewer comment 2.4

SI line 249: you set r_A <= r_B and the assume that I_A, the set of all cells from which a given cell A receives metabolites, is included or equal to I_B, the set of all cells from which a given cell B receives metabolites. Can you explain/justify/demonstrate how r_A <= r_B implies that I_A is included or equal I_B? In the current text there is no explanation about it, or maybe it is distributed in various places.

Response: this assumption is based on a geometric consideration; we have added the following explanation of the underlying reasoning to the SI (line 255):

This assumption comes from a simple geometric consideration: for spherical cells the interaction neighborhood is given by a circle/sphere (in 2D/3D space, respectively) centered on the focal individual with an area/volume that is set by the neighborhood size. The smaller interaction neighborhood is thus always fully contained within the larger one. More generally, for non-spherical cells the interaction neighborhood follows the shape of the focal cell and is defined as the region of space within a fixed distance of the cell-surface such that the total number of cells contained in this regions equals the size of the neighborhood. Cells within the smaller interaction neighborhood are those neighbors that are closest to the focal cell; these cells are thus also members of the larger interaction neighborhood (which extends further away from the focal cell).

Reviewer comment 2.5

Fig 3 caption: "(B) The neighborhood size has only a minor effect on the global composition of the the community". I am not sure I agree with this wording. Even asymptotically there seem to be deviations from the well-mixed system on the order of ~20%? (green lines). Note also the repeated word.

Response: We have rephrased this to:

The neighborhood size affects the global composition of the community, when the two types have different maximum growth rates.
